# The value of water quality for coastal recreation in New England, USA

**DOI:** 10.1017/age.2024.12

**Published:** 2024-11-29

**Authors:** Nathaniel Merrill, Marisa Mazzotta, Kate Mulvaney, Joshua Sawyer, Julia Twichell, Sarina Atkinson, Darryl Keith, Laura Erban

**Affiliations:** 1 Office of Research and Development, Atlantic Coastal Environmental Sciences Division, United States Environmental Protection Agency, Narragansett, RI, USA,; 2 American Forests Inc, Washington, DC, USA; 3 National Oceanic and Atmospheric Administration, Southeast Fisheries Science Center, Miami, FL, USA

**Keywords:** Discrete choice modeling, non-market valuation, recreation, remote sensing, revealed preference survey, water quality, Q50, Q51, Q53

## Abstract

Water recreation is valuable to people, and its value can be affected by changes in water quality. This paper presents the results of a revealed preference survey to elicit coastal New England, USA, residents’ values for water recreation and water quality. We combined the survey responses with a comprehensive data set of coastal attributes, including in-water and remotely sensed water quality metrics. Using a travel cost model framework, we found water clarity and the bacterial conditions of coastal waters to be practical water quality inputs to economic analysis, available at appropriate scales, and meaningful to people and their behavior. Changes in clarity and bacterial conditions affected trip values, with a $4.5 change for a meter in clarity in Secchi depth and $0.08 for a one-unit bacteria change in colony-forming units per 100 ml. We demonstrate the large potential value of improving water quality through welfare analysis scenarios for Narragansett Bay, Rhode Island, and Cape Cod, Massachusetts, USA. The paper discusses lessons for improving the policy relevance and applicability of water quality valuation studies through improved water quality data collection, combined with the application of scalable analysis tools for valuation.

## Introduction

Recreation on, in, and near water provides a physical and emotional connection between people and the quality of surface waters. Research is ongoing to fill gaps in the US Environmental Protection Agency’s (EPA) ability to evaluate policies to improve water quality at a national scale. One identified research gap is the valuation of recreational uses of coastal waters (Keiser et al. 2019), an important and large component of the benefits of coastal water quality improvements. In 2012, the most recent year for which data are available, almost 49 million people in the United States participated in ocean and coastal recreation, encompassing more than 1.2 billion recreation days and $141 billion in direct expenditures. In New England alone, nearly 5.6 million US residents engaged in coastal recreation, including beachgoing, swimming, fishing, shellfishing, boating, and more, with direct expenditures of more than $10 billion (Kosaka and Steinback 2018).

Water quality impacts on coastal recreation have been studied for more than 40 years (Binkley and Hanemann 1978; Strand and McConnell 1985; Bockstael et al. 1987a; Bockstael et al. 1987b; Opaluch et al. 1999; Hilger and Hanemann 2006; English et al. 2018; Phaneuf 2002; Kaoru 1995; Lyon et al. 2018; Parsons et al. 2009; Oh et al. 2008; Lipton and Hicks 2003; Bergstrom et al. 2004). The first application of site-choice modeling in applied environmental economics was an EPA study of beach water quality in the greater Boston, Massachusetts, area (Bockstael et al. 1987a). The many studies on water quality benefits since that time have provided a foundational understanding that there are substantial impacts on the use and value of recreation from changes in water quality. While the focus of policies has shifted over time from addressing point sources of pollution to non-point sources and to different pollutants and impacts, the need for methods and studies to evaluate the recreational benefits of policies to improve water quality remains.

Yet, for many reasons, federal, state, and local decision makers still struggle to quantify recreational benefits. Existing data are limited in geographic and behavioral scope (see Mazzotta et al. 2022 for a literature review). Many studies focus only on open-water beaches (Hilger and Hanemann 2006; Parsons et al. 2009; Oh et al. 2008), although recreation also often takes place in estuaries, lagoons, and other semi-enclosed areas of the coast. In total, these other coastal areas may be used by as many or more people than those who visit nearby open-water beaches (Mazzotta et al. 2022; Mulvaney et al. 2020), and they are more likely to be impaired due to reduced tidal flushing or proximity to sources of pollution. Studies also tend to focus on fishing (Kaoru 1995, Lipton and Hicks 2003, Bergstrom et al. 2004) or beach visitation (Bockstael et al. 1987a; Hilger and Hanemann 2006; Parsons et al. 2009; Oh et al. 2008) though people engage in numerous other coastal recreation activities (Kosaka and Steinback 2018; Mazzotta et al. 2022; Mulvaney et al. 2020; Leeworthy 2001, Leeworthy and Wiley 2001). Additionally, measured water quality parameters and models that reflect what is important and relevant to people and that match the temporal and geographic scale needed for valuation studies are difficult to access and integrate. Together, these challenges limit the ability of EPA and its partners to quantify the benefits of rules and policies that can improve water quality along our coasts, where nearly 40% of the US population lives (NOAA 2021c).

In this paper, we present an application of the travel cost method to estimate the economic value of improving water quality for recreation in coastal areas. Our study covers southern New England’s coasts of Connecticut, Rhode Island, Massachusetts, and New Hampshire, USA, with a focal point of Cape Cod (Barnstable County), Massachusetts. In this densely populated region (population age 18+ years of over 6.5 million), we connected individual choices about where to recreate along the shoreline to water quality and other site attributes at each coastal location. We accomplished this by linking the results of a geographically flexible revealed preference survey to spatially explicit water quality indicators, based on in situ and remotely sensed data. We then estimate a recreational site-choice model using day-trip responses and flexible choice sets of shorelines within 100 miles of the respondents’ homes. In the process, we overcame significant social and environmental data availability and synthesis limitations to produce policy-relevant results that are salient to the public. Our approach can be applied to other regions of the United States or globally to support local or national-scale analyses (Czajkowski et al. 2015).

We provide valuation functions relevant to a wide range of coastal water quality policy scenarios, including non-market economic values per coastal recreation trip for marginal changes in a set of water quality parameters and other site attributes. We demonstrate the application of our models to regional scenario analyses of aggregate economic benefits from water quality improvements to the estuaries on Cape Cod, Massachusetts, USA, and to Narragansett Bay, Rhode Island, USA, illustrating how policy approaches that affect different water quality parameters can be valued. We discuss the challenges of estimating such a model and new steps that could be taken to make the valuation of water quality changes more achievable.

## Data

The data we used in this study are from a survey of households to elicit coastal recreation trip locations and details combined with attributes of the shoreline they could have visited and the nearby coastal water quality. These are described below along with the construction of the choice set for the travel cost modeling. The sources and summary statistics for the shoreline attributes are in Tables 1 and 2.

To ensure the survey questions were understood easily and meaningfully and to collect related qualitative data, we conducted seven focus groups between June and July of 2016 at professional focus group centers in CT, RI, and MA. The focus group participants were members of the public who had engaged in coastal recreation in New England at least once in the previous 12 months. The focus groups were recorded, transcribed, and coded to identify consistent themes. In the focus groups, participants identified a range of locations that they visited for coastal recreation, ranging from smaller fishing access points to large, open-ocean-facing beaches. They also identified a wide range of activities they participated in at these various sites.

From the focus groups, we found that members of the public recognize the term “coastal water quality” as encompassing things in the water that can make one sick and that affect the esthetic experience of visiting the coast, as well as the state of the water for supporting a healthy ecosystem. Respondents in focus groups could consistently identify geographic gradients in water quality that generally match scientific observation. We also found water quality to be a composite idea covering many aspects of the water that affect recreation and the environment. Water quality is complex and sometimes conflicting in some dimensions. For example, the presence or abundance of macroalgae (i.e., seaweed) varies naturally in coastal waters and can be a sign of a healthy ecosystem but can be undesirable for recreation (Hamel et al. 2021). This nuance was not lost on the public in our research, and we found that people are able to hold complex conceptualizations of water quality that encompass multiple components (Artell et al. 2013). Focus group participants consistently identified similar attributes of water that affected their perceptions of its quality: clarity, color, odor, and a history of beach closures (for bacteria). Consistent with the survey findings (Mazzotta et al. 2022), participants identified a number of reasons for choosing a location, with proximity as the most frequently identified and natural features of the site, including water quality, as the second most identified. The focus groups also consistently identified social opportunities with family and friends as well as spiritual and cultural reasons for choosing a location.

### Survey

This study of coastal recreation and water quality in New England captured a diverse set of recreational activities and covered a comprehensive set of coastal sites (see Mazzotta et al. 2022 for a full description of the survey). The study utilized a mail push-to-web survey of coastal recreation and water quality in southern New England, USA, to collect data on respondents’ recreational behavior and the most recent coastal recreation trip in New England within the last 12 months. We conducted the survey in the summer and fall of 2018 and sampled a population within a day-trip distance (100 miles) of Cape Cod, MA. The sample area contains 60% of New England’s total population, including the urban centers of New London (CT), Providence (RI), Boston, Worcester, and New Bedford (MA) (U.S. Census Bureau 2021).

We used a stratified random sample of addresses from the US Postal Service Computerized Delivery Sequence File (DSF), oversampling Barnstable County (Cape Cod), MA, by 3.06 times. We applied a mixed-mode approach with a mailed push-to-web invitation followed by a reminder a week later and then a paper version of the survey two weeks later if we had not yet received a response. We sent the survey to a total of 9,520 addresses. We sent 370 in a pilot in May of 2018, and after deciding to make no changes, we sent it to 9,150 addresses in the full sample in August–October 2018. No monetary incentives were included. Overall, including the pilot and the full sample, we received 1,437 responses, a 15.75% response rate after accounting for undeliverable addresses with 53.6% of respondents choosing to use the web survey version. See Mazzotta et al. (2022) for an extensive summary of the survey instrument, respondents, and summary recreation information. This collection resulted in 1,172 reported coastal recreation trips (882 as part of a day trip, leaving from and returning to home, and 342 as part of overnight trips). For the purposes of this paper, we limited our analysis to the day-trip responses.

As compared with previous travel cost survey work that used limited choice sets, for example, a predetermined set of beaches or lakes, we allowed for a freeform choice set of any coastal location in New England. For online survey responses, respondents specified their trip locations using an interactive mapping function. For paper survey responses, respondents wrote in their trip locations, and we used Google Maps’s application programming interface geocoding search to convert this write-in information to longitude–latitude coordinates (Google 2021). We recovered 706 usable day-trip responses, defined as responses where the inferred location of the recreation trip was within .25 miles of the shoreline and also could be connected to data required to construct travel cost estimates, including shoreline and water quality attributes. See Mazzotta et al. (2022) and the accompanying code and data package (https://doi.org/10.5281/zenodo.5807860) for details.

### Shoreline attributes

The land directly abutting coastal waters in New England varies widely from the fully urbanized Boston Harbor and Providence River to the rural, sandy Cape Cod National Seashore. We used the National Oceanic and Atmospheric Administration’s (NOAA) Environmental Sensitivity Index (ESI) maps and data to capture the types of shoreline that would define possible coastal recreation areas (NOAA 2021b). Originally designed to gauge the risk from oil spills to the natural and human-use resources of the coastal area, NOAA’s national data set segments the shoreline by types (i.e., sandy, rocky, vegetated, armored), slope, and whether it is exposed to or sheltered from wave energy.

**Figure 1.**
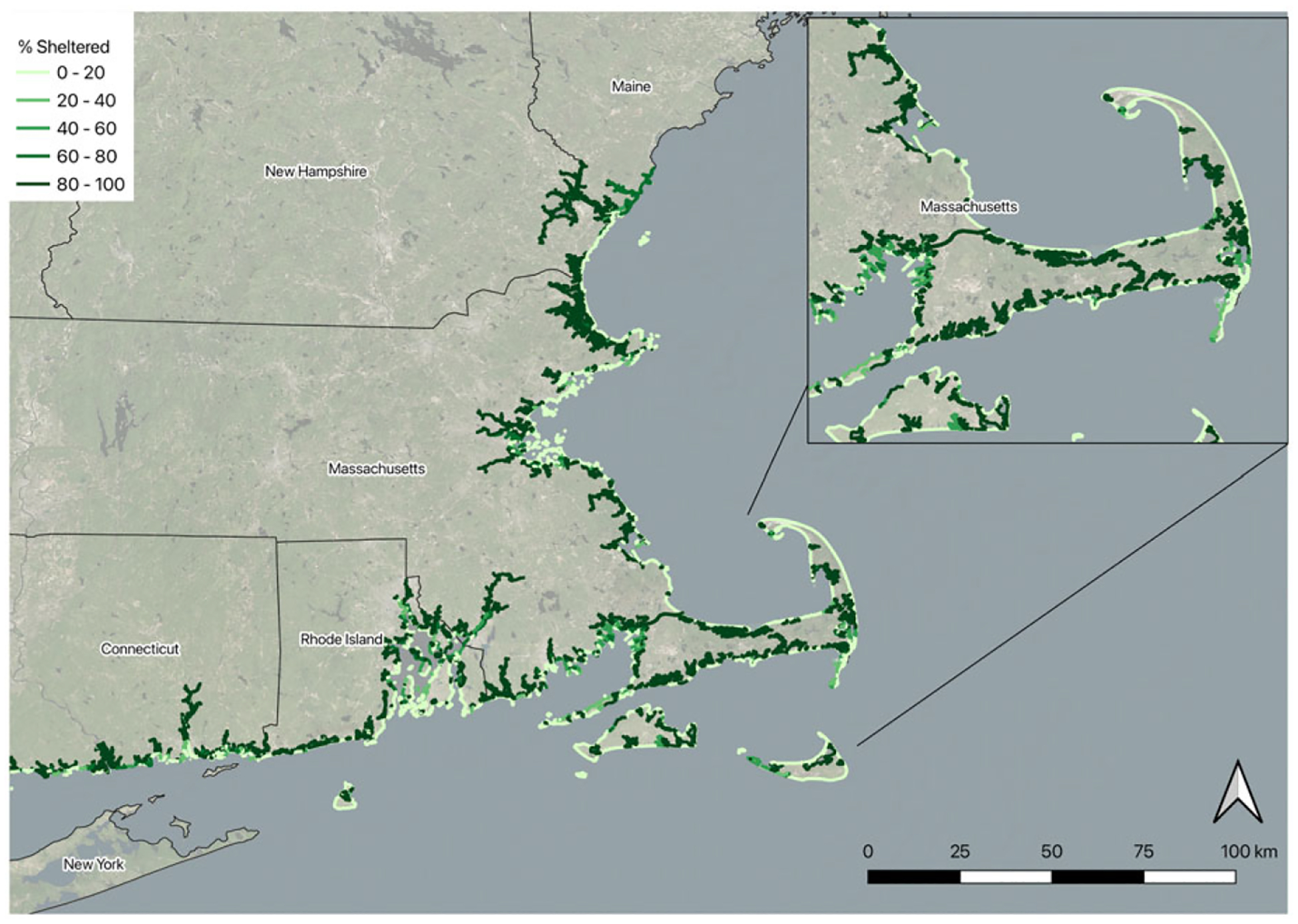
NEWQ shoreline segments used as the choice set in the RUM model with one of the attributes, “sheltered from wave action,” displayed.

We merged the ESI GIS data for each of the New England states into a single data set and simplified the geography by removing any lines that were not categorized as shorelines, which include docks, piers, jetties, groins, and breakwaters. We then created simplified shoreline types in four categories (sandy, rocky, vegetated, armored) based on the ESI data and dissolved adjacent shorelines of like categories. We also retained the exposed or sheltered characteristic (Figure 1, see Supplementary Materials for ESI shoreline type crosswalk and additional maps showing shoreline segments).

We retained the length of each shoreline segment. In sinuous shoreline locations, such as in marshes or areas with small peninsulas, the shoreline segment length might be much greater than the straight-line distance along a water body. This straight-line distance may be a better measure of the geographic scale people perceive. Due to the coastline paradox, a shoreline’s length is different depending on the scale of analysis or cartographic detail (Mandelbrot 1982), so there is no definitively correct length measure. Therefore, we created a second length attribute that provides a more comparable measure across shoreline segments by measuring the diameter of a minimum bounding circle that encompasses the entire segment: the “straight-line length” (Gomez et al. 2017).

A great deal of coastal recreation in New England occurs at beaches. Under the Beach Environmental Assessment and Coastal Health Act (BEACH Act; 33 U.S.C. §1313 et seq. 2000), the US EPA provides grants to assist local authorities in monitoring their coastal beaches and issuing notifications to protect the public from unsafe conditions. The Beach Advisory and Closing Online Notification (BEACON) system then provides a database of beach closures and conditions reported by states. As part of that database, the locations of monitored beaches are provided including lines representing the beach area (EPA 2021). We used these beach lines to indicate whether our simplified shorelines included designated beaches.

We found from our focus groups that the land area adjacent to the coastline affects people’s perceptions of the quality of the area. The naturalness, or amount of development nearby, was a factor people considered when choosing where to recreate. It is also correlated with the water quality variables that we intended to study. To characterize the naturalness of the area we summarized the impervious surface within a ¼ mile buffer of each shoreline segment. We used the National Land Cover Database (NLCD) 2016 Percent Impervious data set and created the percentage of impervious surface within the buffer excluding the water pixels to capture the characteristics of the land nearby (Dewitz 2019).

We processed the shoreline data using ArcGIS ArcPy scripting functionality (ESRI 2020). This simplified shoreline data set, hereafter New England Water Quality (NEWQ) shorelines, resulted in 17,665 shoreline segments from Connecticut to Maine with attributes of shoreline type, wave exposure, beach designation, length, and nearby land use.

### Water quality

In valuing water quality changes in a revealed preference study, ideally, the multiple interrelated components of water quality would be captured together. Water quality index concepts have been proposed to try to capture the multi-dimensional nature of water quality. Practically, however, creating an index of more than a few parameters that can be applied consistently to many locations proves challenging, given data and model availability, especially for coastal waters. There are few comprehensive landscape-scale summaries of water quality well matched to the type of metrics and geographic and temporal scales needed. The roots of the problem in coastal waters are a sparse observational network, a lack of harmonization of collection methods, and poor data standards and storage with varying degrees of sharing and discoverability. To overcome these challenges, much of our research effort focused on the creation, compilation, and linking of water quality metrics to match the scale of human decisions (i.e., individual coastal access points) and the scope of a regional analysis.

**Figure 2.**
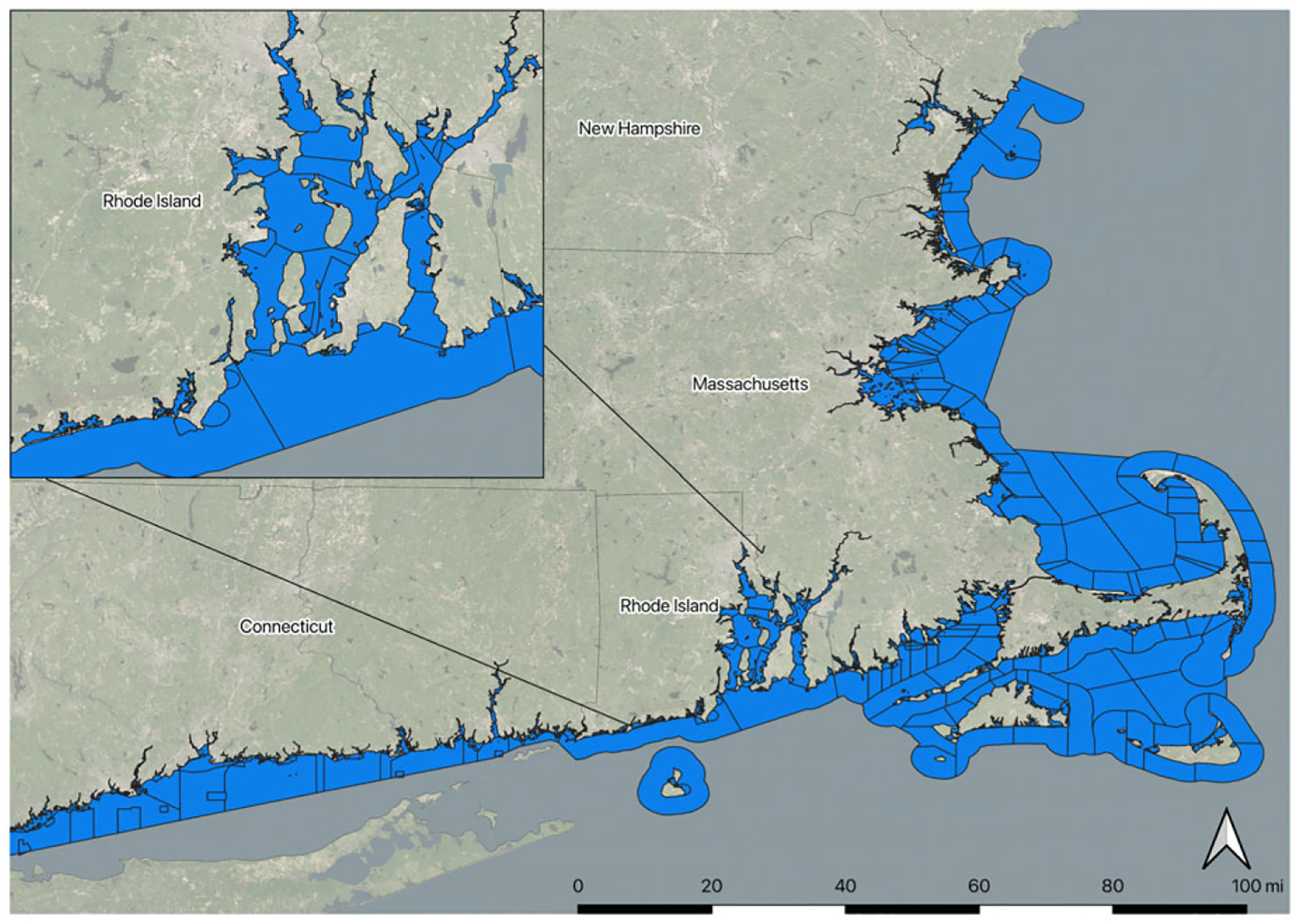
NEWQ water segments of coastal waters used for summarizing water quality metrics.

Having already segmented the shoreline, we segmented the coastal waters, in order to match water quality metrics to shoreline recreational locations. The nearshore waters in New England vary widely, including open ocean, lesser-developed and highly developed estuaries, and urban harbors. This variety offers conditions for a range of both recreation activities and water conditions. Water quality, the primary interest of this study, also varies with different landward inputs and amounts of ocean tidal influence. Most of the New England states’ 303(d) impaired waters lists under the Clean Water Act divide areas of coastal water into management units that reflect geography and hydrology as well as policy and human influence, such as wastewater treatment outflows. Three of the sampled states (Rhode Island, Massachusetts, and New Hampshire) use similar coastal water divisions for their shellfish advisory and closure programs. Connecticut uses different divisions for their shellfish program and their 303(d) program and their segmentation for their shellfish program matched the other states’ better than their 303(d) impairment units. Therefore, we combined Connecticut’s shellfish management coastal water segmentation with the other states’ 303(d) listing segments (see Table 1 for data set references). We excluded Maine from this study given the unavailability of consistent water segmentation. We merged the other New England states’ coastal water units into a comprehensive set for our waters of interest, which we refer to as “NEWQ water segments,” comprising 1732 segments (see Figure 2). We then connected these water segments and their 303(d) impairment status to their adjacent shoreline segments with multiple shoreline segments matched to their corresponding water segment.

To synthesize in situ water quality sampling data, we queried the Water Quality Portal (WQP), a national repository of water quality data from multiple agencies and partner organizations (Read et al. 2017). We cast a wide net for any in-water marine samples for New England from 2000 to 2020, resulting in millions of records from more than 50 organizations. While the Water Quality Portal data covered a portion of the time and space we needed, some of the largest programs in the region, such as the Buzzards Bay Coalition’s monitoring program, were not included in the Portal at the time of retrieval. Therefore, we combined the Water Quality Portal data with data obtained directly from the Buzzard’s Bay Coalition and from a data set covering multiple groups built by the Massachusetts Department of Environmental Protection and EPA’s Office of Water for nutrient criteria analysis in estuaries. We scripted the process in R 4.1.1 (R Core Team 2013). This helped cover missing areas of interest around Cape Cod, Massachusetts.

We narrowed the in situ water quality data set of interest down to water clarity (Secchi depth in meters), surface chlorophyll *a* (Chl *a*; mg/l), and Enterococcus as measured by colony-forming units (CFU) or most probable number (MPN) per 100 ml taken in summer (June–September). Enterococcus is used in marine waters as an indicator of contamination by fecal matter and the presence of other harmful bacteria, viruses, and protozoa (EPA 2023a). It is the most commonly measured indicator for marine beach water quality (EPA 2023b). These sample events were associated with the NEWQ water segments in which they were taken. We then made statistical summaries (mean, median, max, min) of these chosen metrics for the water segments over chosen time windows. While there are numerous other types of water quality samples taken in the region (e.g., nutrients, pH, dissolved oxygen), we selected these particular metrics for two reasons. First, the identified metrics are those most relevant to how the public considered coastal water quality based on our focus group and survey responses. Second, the need for widespread and frequent measurements over space and time for our analysis led us to these more commonly sampled water quality metrics.

This query revealed big gaps for usable in situ water quality sampling data in much of New England (Merrill et al. 2021). Setting even a loose criterion of at least 10 samples over the last 10 years (2010–2020) for each metric in a NEWQ water segment resulted in only 42 out of 1732 (2%) of the segments meeting that criterion for having enough Secchi measurements, 5% for surface Chl *a* and 15% for CFU or MPN measurements. Data sharing, compiling, and reporting are ongoing issues in New England (and nationwide) coastal waters, where data are scattered across numerous organizations and measures are inconsistently taken, recorded, and housed. Improving data standards and warehousing would improve our collective understanding of coastal water quality greatly.

Some existing federal policy partially bridges this gap by requiring bacterial sampling at swimming beaches. Monitoring through the BEACH Act has resulted in by far the most spatially comprehensive and consistent indicator of water quality in the coastal zone that is relevant to people. The BEACH Act’s requirements to collect a relatively standardized measurement in many places and the clear connection to human health and beach closure policy lead to useful data toward understanding water quality’s effect on recreation at monitored beach sites. This beach water sampling housed in the WQP is the source of most of the Enterococci CFU or MPN measurements for our data set. While this monitoring is reasonably comprehensive across swimming beaches, it does not necessarily provide information on non-beach access points, which represented 30% of the coastal recreation trips in our survey.

**Figure 3.**
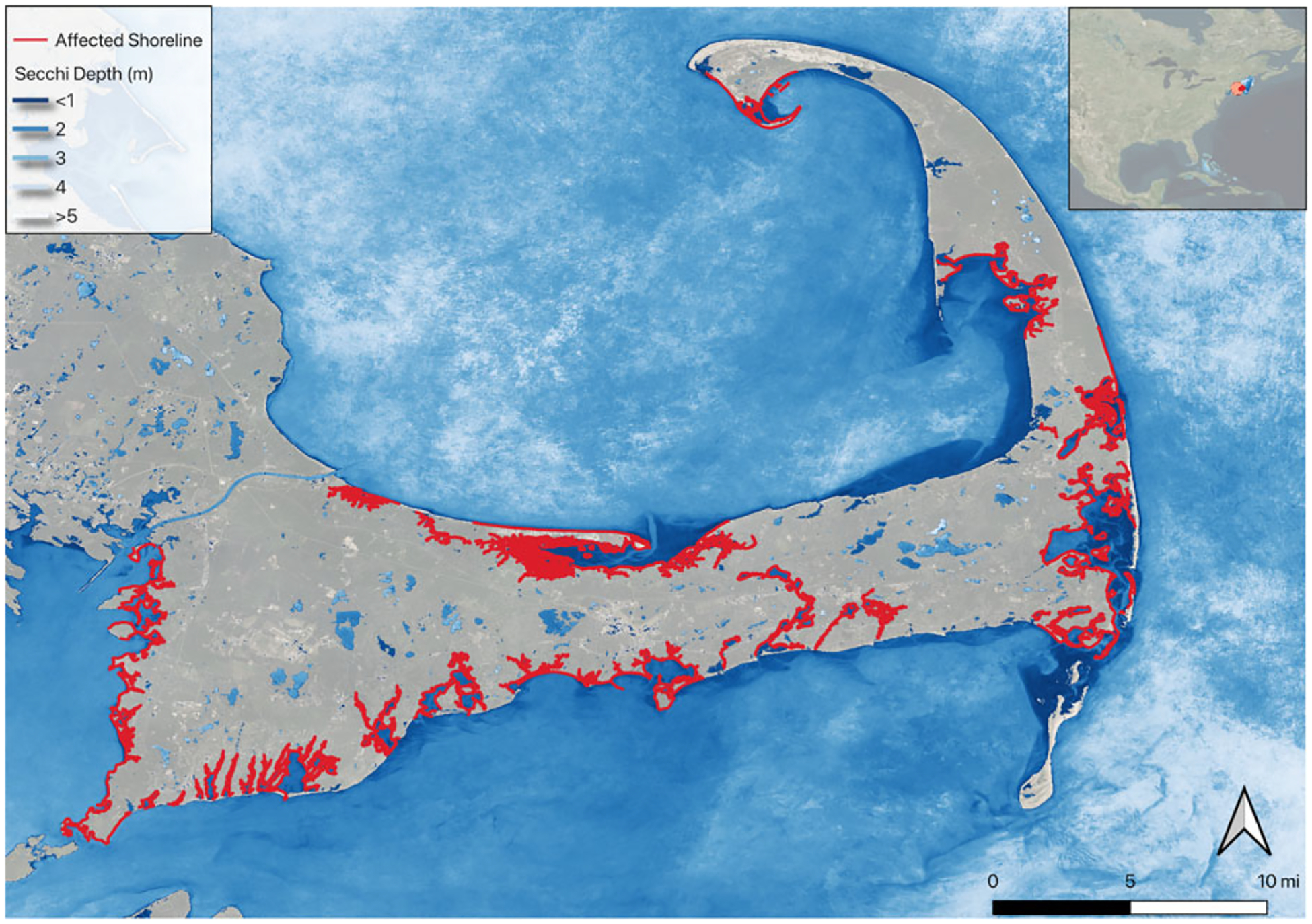
Cape Cod, MA, showing water clarity and the shorelines with changes in clarity for the welfare scenarios.

**Figure 4.**
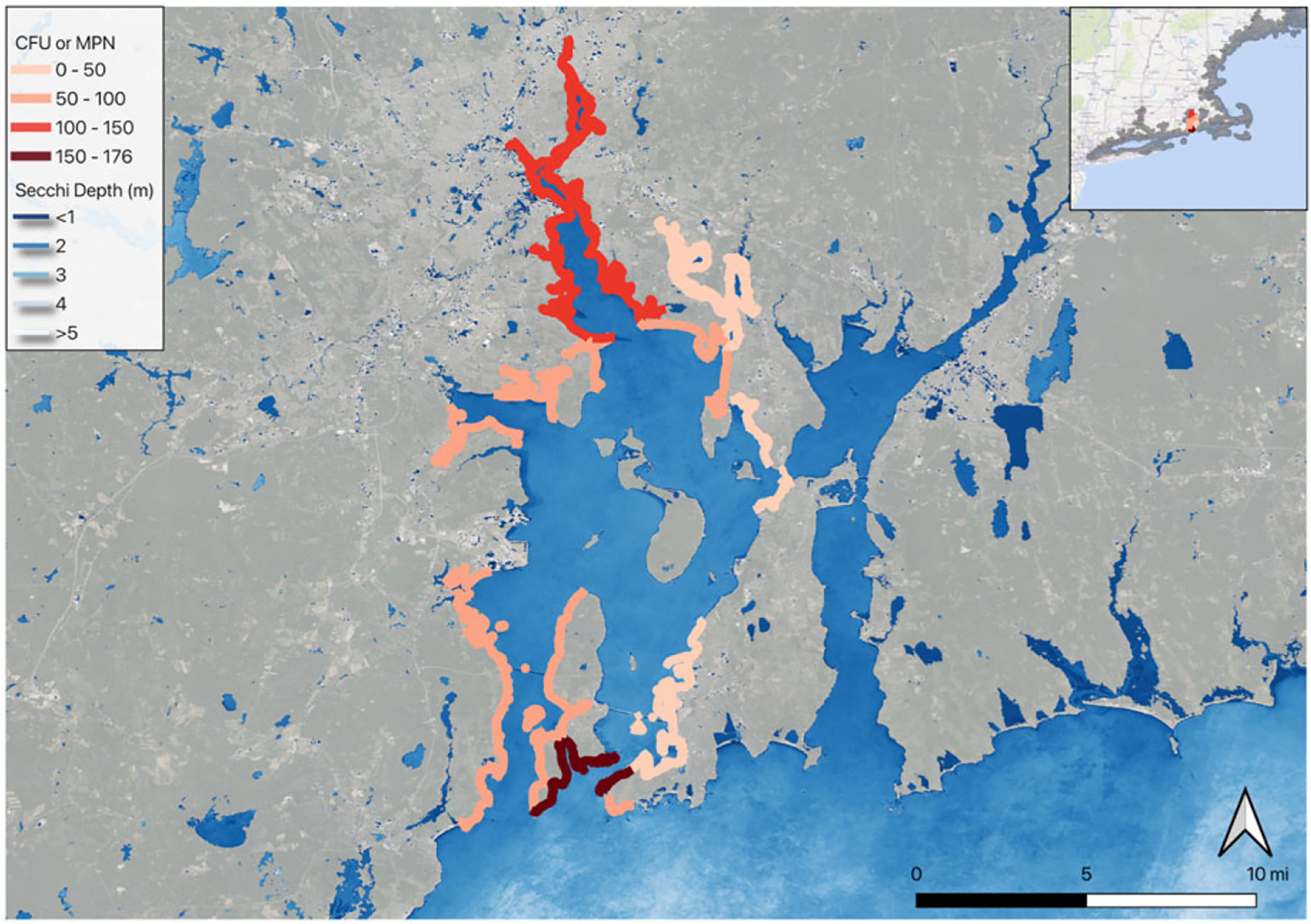
Narragansett Bay, RI, showing baseline bacteria conditions on the shorelines with changes in the welfare scenarios.

Due to these limitations in the in situ data sets and to provide a more comprehensive view of coastal water quality, we developed an additional water quality measurement using remote sensing. We produced a 30-meter resolution raster of Secchi depth for coastal and estuary waters by applying a quasi-analytical algorithm (Lee et al. 2016) in Google Earth Engine to data from the NASA/USGS’s Landsat 8 OLI instrument (see Google Earth Engine code reference in Table 1 and rendering in Figures 3 and 4). This algorithm has shown promise in lakes as well as coastal waters (Luis et al. 2019). The algorithm we coded is flexible and can create pixel-level summaries for certain time windows and months of interest. We created summaries of Secchi depth representing median summer conditions (i.e., median summer Secchi depth) for the sensor record (2014–2018 for Landsat 8). We connected this summer median condition raster to the NEWQ shorelines using a ¼ mile buffer from each shoreline segment and summarized the pixels (mean, median, max, min).

### Choice set and travel costs

For the choice set for the site-choice model, we matched the NEWQ shorelines with the water quality from the adjacent NEWQ water segments and remote sensing attributes. After removing locations in Maine and shoreline segments not adjacent to coastal water segments (far inland river segments), 7,243 shoreline segments were left in the overall choice set for the random utility model (RUM). Bacteria measures were only available for a subset of water segments and therefore a subset of shoreline segments. The segments with bacteria measures are mostly in water quality segments that contain beaches, given the beach monitoring programs (Table 2).

We connected each survey trip response to the shoreline segment closest to the trip location identified by the respondent if the chosen location was geolocated within ¼ mile from one of our shoreline segments. We calculated the distance and travel time from each respondent’s home census block where the survey was mailed to each choice alternative (shoreline segment) through a road network using an Open-Source Routing Machine (Luxen and Vetter 2011). This created a standard site-choice model data set where the location chosen by the respondent and its attributes can be compared against all the alternative choices for that respondent within 100 miles of their home census block. This resulted in varying individual choice sets ranging from 999 to 5,956 shoreline segments.

From the calculated time and distance matrices, we created the travel cost for each respondent to visit each shoreline segment using the following equation (English et al. 2018):

(1) $$T_{in}=2\left[\frac{g*\text{mile}s_{in}}{\text{adults}}+\left(\frac{1}{3}*\frac{\text{incom}e_{n}}{2080}*\text{hour}s_{in}\right)\right]$$

where:

*T*_*in*_ = round-trip travel cost of for respondent *n to* shoreline *i*

*g* = fuel cost, depreciation, and maintenance per mile – 28¢ per mile^1^

*miles*_*in*_ = one-way travel distance in miles for respondent *n* to shoreline *i*

*hours*_*in*_ = one-way travel time in hours for respondent *n* to shoreline *i*

*income*_*n*_ = yearly household income in USD for respondent *n*. This is divided by 2,080 (the standard number of work hours in a year).^2^

*adults* = average adults on reported trips – 2.4 adults

## Methods

We estimated the effects of water quality on coastal recreation values using the cross-sectional (spatial) variation in 5-year (2014–2018) average summer water quality conditions across the many shoreline segments in the choice set. Following the theory of the RUM, we used discrete choice conditional logit models to estimate the log odds of a respondent choosing a site (shoreline segment) as a function of the site attributes, water quality measures, and travel cost (Phaneuf and Smith 2005; Parsons 2003 and 2017). These are fit using maximum likelihood estimation. See Supplementary Materials for an explanation of the RUM theory in this case and its relationship to the logit model and choice probabilities.

We estimated a conditional logit model specification, as follows, using only day-trip responses:

(2) $$v_{\text{in}}=\alpha \;T_{\text{in}}+\zeta X_{i}$$

where:

*v*_*in*_ = utility for respondent *n* visiting site *i*

*α* = the travel cost coefficient or marginal utility of the cost of the trip

*T*_*in*_ = cost of choosing site *i* for respondent *n*, the travel cost

***ζ*** = a vector of coefficients for site attributes, ***X***_*i*_

We estimated the logit model in R 4.1.1 using the Rchoice package (R Core Team 2013, Sarrias 2016. See code package for the scripts). For further explanation of the conditional logit as applied to RUMs, see Parsons (2003). Since we had missing values for CFU or MPN for some shoreline segments, we assigned a constant, zero, for missing values and included a dummy variable for the presence or absence of this measurement. Although a straightforward and common method for dealing with missing values, this may induce bias in coefficient estimates (Jones 1996). As a robustness check, we include a model in Table 3 using a subset of the data with only trips reported to beaches, which resulted in a data set with completeness in the bacteria variable.

There are a wide variety of discrete choice model estimation methods that have been applied in recreational demand applications. In the Supplementary Materials, we show the results of a two-stage alternative specific constant (ASC) model applied to simplified choice sets and a mixed (random parameter) logit. For the ASC model, we estimated a specification with ASCs and employed a second-stage regression to recover marginal utility estimates for site attributes, an approach proposed to control for omitted variable bias and differences across respondents in recreation demand models which may affect identifying the travel cost coefficient (Murdock 2006; Abidoye et al. 2012). The mixed logit model assumes each respondent’s parameters may come from a distribution instead of a single value, allowing it to capture more flexible choice patterns than the assumptions needed for the standard logit model (Train 1998). However, to run these logit variations, other limitations were introduced. For these variations, we were required to simplify our data by aggregating sites or dropping alternatives, which obscures the disaggregated site conditions we sought to study. These also needed additional statistical modeling assumptions, which may result in their own biases (Domanski and von Haefen 2018; Greene and Hensher 2003; Lupi and Feather 1998).

### Welfare estimates from regression and water quality scenarios

We estimated marginal effects, or changes in the value of a trip to an average location given an attribute change, by dividing the coefficient of a shoreline attribute, *ζ*, by our estimate of the marginal value of income, *−α*, the coefficient on the travel cost variable. Costs are entered into our data set as positive numbers, so the marginal value of income is the negative of the travel cost coefficient.

To illustrate how these results can be applied at the aggregate level to policy questions for benefit-cost analysis, we developed two scenarios related to current policy efforts to improve coastal water quality within the sampled New England region and within our choice set in the behavioral model. We created a scenario of improved estuary water clarity on Cape Cod, Massachusetts, and a scenario of bacteria condition improvements in Narragansett Bay, Rhode Island. To create the welfare scenarios, we changed the water quality attributes for associated shoreline segments (i.e., the choice locations) in the choice set within each scenario’s geographic scope (see Figures 3 and 4). While water quality models for both Narragansett Bay and for estuaries on Cape Cod exist, they do not currently simulate outcomes appropriate for valuing the recreational benefits of interventions being conducted or under consideration in their watersheds neither in the water quality metrics being simulated nor in the spatial and temporal extent desired. Therefore, we present the welfare scenarios as a range of possible improvements in percentage terms. We applied a change in a single water quality metric to each scenario although, in reality, clarity, bacterial, and other conditions could all improve concurrently as a result of policy-driven interventions, leading to greater benefits than our estimates show. However, we do not know how these metrics of clarity and bacteria would change together specifically for our region or policy scenario. Additionally, we held the other landscape inputs fixed (impervious surface, for example), but these may also change depending on the steps taken to achieve water quality improvements.

The towns on Cape Cod are in the process of creating and implementing plans to improve water quality in the many estuaries on the sandy peninsula. Nutrient pollution has led to a decrease in water clarity and impaired benthic conditions due to excess algal growth (Cape Cod Commission 2015). The costs of addressing this nutrient pollution include those associated with new sewer lines and enhanced centralized and individual wastewater treatment as well as a variety of alternative methods to limit nutrient impacts in the estuaries (Perry et al. 2020, Merrill et al. 2021). The social benefits remain largely unquantified, though recreation is likely to be one of the larger categories of benefits due to Cape Cod’s recreation and tourism-based economy (Cape Cod Commission 2015). For Cape Cod’s scenarios, we chose the segments associated with all the estuaries and embayments on Cape Cod. We did this selection manually in QGIS (QGIS.org 2021). To estimate values for increased clarity for those shorelines we applied a uniform percentage increase (5%, 10%, 20%) to the Secchi depth estimate for each shoreline (see Figure 3).

Through a multi-state effort spanning several decades, water quality conditions in Narragansett Bay may be improving. Stormwater runoff, combined sewer overflows, and development have decreased Narragansett Bay’s water quality for habitat, recreation, and shellfishing. Significant improvements in infrastructure have been made, including the construction of a series of large underground stormwater storage facilities (combined sewer overflow tunnels) to capture polluted runoff and upgrades to wastewater treatment plants. Monitoring changes in water quality and other environmental conditions is ongoing (NBEP 2017).

Narragansett Bay contains a number of beaches and swimming locations that are periodically closed due to excess bacteria. For the Narragansett Bay scenarios, we chose segments starting with the Providence River to the north, following the western portion of Narragansett Bay to its southern mouth on the Atlantic Ocean. The scenario segments are bordered on the west by the full western shoreline of Narragansett Bay and on the east by East Providence, Barrington, Warren, Bristol, and Aquidneck Island (Portsmouth, Middletown, and Newport). We did not include shorelines in the Seekonk River, Mt. Hope Bay, and Sakonnet Passage, which may be hydrologically more disconnected from upgrades to infrastructure in Providence. We did this selection manually in QGIS. Shoreline segments with no existing CFU or MPN data were also excluded. We applied a uniform percentage decrease (5%, 10%, 20%) in the CFU or MPN estimates for shorelines that had those estimates available, mostly those near beaches (see Figure 4).

We used the logit model (equation 2) results to predict the baseline and new utility of visiting each site for each respondent. To calculate changes in per-trip consumer surplus resulting from water quality improvements, we use the log-sum values for each respondent to create differences in welfare from before to after the changes (Parsons 2017).

(3) $$\Delta CS_{n}=\frac{\text{ln}\left[\sum_{i=1}^{I}e^{{v_{in}}^{1}}\right]-\text{ln}\left[\sum_{i=1}^{I}e^{{v_{in}}^{0}}\right]}{-\alpha }$$

where:

Δ*CS*_*n*_ = change in consumer surplus for respondent *n* between scenario 0 and 1

*v*_*in*_^0^ = fit values from equation 2 for each site for scenario 0, or scenario 1, for site *i* and respondent *n*

*α* = the travel cost coefficient or marginal utility of the cost of the trip

We created these differences in log-sum values for each individual and then averaged over individuals using the survey weights to correct for the geographic oversample for Cape Cod to create population-level estimates of per-trip, per-person willingness to pay changes due to changes in site attributes. We then applied these per-trip changes in willingness to pay to the total number of summer trips from our sample region (80M) as estimated by self-reported frequency (see Supplementary Materials Table S3).

We created net present values of each water quality improvement scenario assuming a consistent per-year benefit and

(4) $$\text{NPV}=\frac{B}{r}$$

where:

*NPV* = net present value of a stream of benefits

*B* = yearly $ benefits of welfare scenario

*r* = discount rate – 3% for our application

We created standard errors of the welfare estimates using the Krinsky–Robb method, sampling 1,000 coefficient sets from their joint probability distribution from the fit logit model, creating the welfare metrics of interest (e.g., marginal effects or aggregate WTP) using each coefficient set draw and then summarizing the resulting empirical distributions of the welfare metrics (Krinsky and Robb 1986). We simulated this process in R. See code package and scripts.

## Results

Table 3 presents three resulting models. Model 1 includes all survey observations and water clarity as the water quality data input. Model 2 includes all survey observations, water clarity as well as bacterial conditions using a dummy variable to control for sites where the in situ data are available. Model 3 subsets the sample to only those survey observations for trips to designated beaches and limits the choice set to designated beach locations. Trips to designated swimming beach locations were worth $25 more per day than those to other shoreline types (Model 2). However, 30% of trips were not to designated swimming beaches. Therefore, ignoring the effect of water quality on recreation at non-beach shorelines excludes a large portion of coastal recreation values in benefits analysis, particularly when considering that sites with degraded water quality are less likely to be designated swimming beaches.

The marginal effect estimates show that water clarity influences people’s choices for trip locations: a 1-meter change in clarity is associated with a $4.5 change in the value of a trip. The bacterial conditions in nearby waters also matter to site choice and economic value, with a $0.08 change in value for a 1-unit change in the CFU or MPN conditions. The coefficient of the binary variable indicating the impairment status of the nearby waterbody implies that impaired waters are more likely to be visited, controlling for the other water quality factors we included. While counterintuitive, this may indicate the locations that are more valued and visited by the public are more likely to be evaluated for impairments.

To put these results in perspective, in previous work (Lyon et al. 2018), we conducted a meta-analysis of beach and swimming day values using 25 studies from across the United States and adding beach length and closure history for the included beaches. The meta-analysis estimated a WTP per trip of $23 per day for a beach in New England with past closure history and $50 per day for New England beaches with no past closure history (in $2018). Comparing the effects of marginal changes in water quality on coastal recreation, which we estimate here, to past work is more difficult because there is little comparable work available. The most comparable study is from the Peconic Estuary (Opaluch et al. 1999), conducted in 1995, where the authors estimated a per-trip increase in WTP of $1.52 ($2018) for a 10% increase in all water quality parameters in the estuary (nitrogen, coliform, brown tide cell counts, and Secchi disk depth) for swimming trips only. Phaneuf (2002) estimated water quality benefits for North Carolina using a model aggregated to the 8-digit hydrological unit and data from the 1994 National Survey of Recreation and the Environment combined with EPA’s Index of Watershed Indicators. They estimated benefits for a scenario where “a maximum of 10% of monitoring station readings for all pollutants included in the model are out of criteria (p. 9).” This resulted in a mean WTP of $0.17 per trip. Because of the way results are reported and the variations in geographic scope in these and other existing studies, it is not possible to directly compare these results to those from the current study.

### Cape Cod, MA

We estimated aggregate seasonal economic benefits from improved clarity in Cape Cod’s estuaries using the standard tools of welfare analysis from discrete choice models explained in the methods. We applied clarity improvements of 5%, 10%, and 20% to shoreline segments associated with Cape Cod’s estuaries and estimated welfare changes using our valuation functions (Figure 3 and Table 4, Model 2). These improved conditions would result in increases of $0.02, $0.03, and $0.07 per person in the per-trip value of recreation for the population in our sample region. It represents the improvement in the respondent’s choice set and expected value of a trip. Therefore, this seemingly small improvement is applicable to all trips taken from the region. While it is plausible that improvements in water quality could lead to more trips in total, we conservatively assumed the total number of summer coastal recreation trips remain the same from our region (80 million trips/year) and applied the per-trip improvements. This led to aggregate annual benefits of $1.3 million, $2.6 million, and $5.2 million for the three levels of clarity improvement. This translates to discounted present values of $43 million, $86 million, and $175 million for improvements to estuaries on Cape Cod, using a 3% discount rate and assuming the water quality improvements continue permanently.

### Narragansett Bay, RI

We valued improvements in bacterial conditions in a scenario analysis that applied a 5%, 10%, or 20% improvement in bacterial levels (measured by CFU or MPN) for shoreline segments that we attributed as being affected by these conditions: mostly shorelines with swimming beach areas where we had bacterial measurements (see Figure 4). We found increases of $0.02, $0.04, and $0.09 per person per trip for the 5%, 10%, and 20% improvements in bacterial levels, and annual aggregate value for all trips in the region of $1.8 million, $3.6 million, and $7.2 million. This translates to discounted present values of $59 million, $118 million, and $242 million for improvements to waters in Narragansett Bay assuming a 3% discount rate (Table 4).

### Alternative model results

As discussed earlier, we also ran alternative discrete choice model specifications proposed in the recreation demand literature: ASC models and mixed logit regression. The mixed logit regression resulted in somewhat lower values for water clarity impacts ($2.7 vs. $4.5 per meter) and for bacteria conditions ($−.06 vs. $−.08 per CFU). The travel cost coefficients from the ASC models were similar to those from the standard logit models presented above (−0.061 vs. −0.051). The ASC results for bacteria conditions were consistent with those from the logit model, but water clarity was not significant in the ASC model. The ASC model, applied to this cross-sectional data, is designed to avoid bias in the identification of the travel cost coefficient value and resulting bias in the marginal effect and welfare estimates that are derived using it. So, the ASC model framework provided a reasonable robustness check for the travel cost coefficient that we found in the standard logit model. However, the simplifications and aggregations we needed to make to the choice set to run the model limited the findings of this work in important ways. First, the aggregation of site-level water quality into large geographic units (county level) obscured insights into site-level behaviors (Parsons and Needelman 1992). Second, to fit the constants, the choice set had to be limited to locations chosen by survey respondents, eliminating all other possible substitute sites. Similarly, the mixed logit model added statistical complication with limited qualitative differences in the findings (see Supplementary Materials).

## Discussion

We found that southern New England residents greatly value coastal recreation and that values are higher for locations with better water quality. The models we created provide non-market values for coastal recreation for the region, encompassing all activities and locations. These estimates were missing to date across this large and highly populated region with numerous coastal access points. The models provide estimates of how the values would change with changes in policy-relevant dimensions of water quality – bacteria and clarity – in the range of waters that are affected by water-improving policies. These policies include, for example, stormwater control efforts, wastewater planning, and swimming beach management, making the results practically useful for many valuation contexts.

Our survey and travel cost model extends the valuation of coastal recreation to multiple access types across a wide geography. Online survey mapping functionality provided us the flexibility for respondents to specify trips to anywhere on the coast rather than being limited to a predefined set of locations as in existing studies (Hilger and Hanemann 2006; Parsons et al. 2009; Oh et al. 2008; Effimova 2019). We did this intentionally to capture a wider range of water recreation activities and places that may be most affected by the water quality policy (Lupi et al. 2020). Near-coastal residents have numerous choices of access points, and our focus groups, survey, and other research (Mulvaney et al. 2020; Merrill et al. 2020) showed that while designated beaches and fishing access points are popular, people visit many places along the coast that typically have not been included in studies that specify locations for the choice set. These include smaller sites closer to urban areas that may be most accessible to communities of environmental justice concern.

In the past, computational limitations affected what was practical in terms of the size and resolution of the choice set. Our study design required compiling landscape-level attributes, like water quality, at a high spatial resolution that we could connect to a large set of coastal areas. We overcame these challenges using modern GIS analysis and remote sensing processes (Gorelick et al. 2017; Google 2021; Luxen and Vetter 2011; QGIS.org 2021). Combined, these tools support scalable and generalizable workflows for fitting and applying valuation models. By creating comprehensive and fine-scale water quality inputs, we were able to assess the variation in water quality at a human-relevant scale for the choice model.

There is a critical need to measure water quality parameters and simulate conditions that are consistent with economic valuation concepts. Remote sensing products show promise for providing water quality information of sufficient coverage and resolution for economic and other place-based valuation studies. As those data, algorithms, and products have become accessible, so too have their applications (Wolf and Kemp 2021; Kuwayama et al. 2017; Franco and Maconald 2018; Zhang et al. 2022; Jain 2020). As the ability to characterize water quality remotely improves with higher resolution and frequency of spectral and other data types, remote sensing will continue to be an important tool for augmenting and scaling our observation systems. While the accuracy and bias of remote sensing data can be estimated through comparisons to in situ water quality measurements (Luis et al. 2019), more work is needed to address both how accurately remote sensing products capture in-water conditions and how well they capture the aspects of water quality that people value at the appropriate scale. We have little understanding of how well remote sensing-derived products or spatial and temporal summaries of in situ measurements capture people’s perceptions of water quality in a waterbody or along a shoreline. The question of how specific and highly resolved in space and time water quality information must be to best match people’s perceptions and predict their behavior remains to be addressed. Our focus groups and survey collected qualitative and quantitative data on water quality perceptions, and future work will address which water quality measures and approaches to summarization most closely reflect perceptions and values.

Coordinating in-water sampling and improving data reporting standards and requirements is critical to improving the accessibility and usability of water quality data for researchers and the public (Sprague et al. 2017, Egan et al. 2009). The National Water Quality Portal provides some standardization and centralized data access but submitting data is voluntary and therefore inconsistent. Data remain sparse, scattered, and difficult to discover, limiting scalable applications for valuation. Our extensive effort to overcoming these challenges provides practical ways forward and spotlights a mismatch between how the public conceptualizes and values water quality and how specialists monitor and model it, which undermines benefits assessment.

Regarding social data, while revealed preference economic valuation methods are well-developed and continuing to improve, their application remains limited by the effort and expense of implementing surveys. National-scale surveys that include questions on recreational choices and water quality perceptions for a full range of recreational activities and types of locations are needed to better address water quality valuation. This might be accomplished by a revival of the National Survey on Recreation and the Environment’s coastal module or a similar periodic data collection akin to NOAA’s National Marine Fishery Service surveys (Leeworthy et al. 2005, NOAA 2021a). A series of intercept surveys in locations rich in water quality data could help improve our understanding of water quality perceptions, appropriate water quality metrics, and other related questions.

Because survey response rates are falling (Stedman et al. 2019), surveys need to be implemented in ways that capture the perspectives of diverse groups of people and samples of sufficient size. Community science approaches might be further developed to address some of these challenges. In addition, the development of passive methods using new sources of data, such as cell phone location data, can contribute to future research on recreational values (Merrill et al. 2020). Finally, research is needed to address environmental justice considerations related to recreational access and water quality, including capturing a broader diversity of people in data collections and highlighting locations where access and quality of experience are not equitable. Visitation and associated demographic data can provide relevant information for assessing other social questions, including those related to the distributional effects of policies (Twichell et al. 2022).

## Conclusions

Large gaps and research challenges remain in understanding how water quality affects the use and values for coastal recreation, despite over 40 years of research attention and general acknowledgment of its importance. This leaves policy analysts and decision makers at the local to national level without critical information to inform decisions. These issues are not unique to the United States. In this paper, we described some of the critical issues and our approach to addressing them. The application of revealed preference valuation methods to water quality valuation for both recreational values and property values remains limited by the scarcity of useful observations and models of water quality that match what people care about for the many locations and activities that people enjoy in coastal waters. Our approaches to address this issue led us to develop ways to harmonize and combine multiple sources of water quality data over a relatively large region of the New England coast.

This work can be built upon in future research and implementation, including (1) developing and applying survey methods to capture a large, fine-scale choice set and any recreational activity; (2) resolving issues related to linking in situ and remotely sensed environmental data to people’s choices and values; (3) collecting recurrent, relevant social data at a national scale; (4) developing survey methods to better capture a diverse and sufficiently large sample in the face of declining response rates for traditional methods; (5) developing consistent and proven methods for using newer social data sources; and (6) developing methods and data collections to address environmental justice concerns related to recreational access and water quality.

Coastal recreation provides great value to southern New England, and that value can increase with water quality improvements. In the scenario for Cape Cod, we estimated values for incremental improvements in water quality of over $100 million for improvements in water clarity that could result from reducing nutrient loading. For Narragansett Bay, reductions in bacterial loading resulted in values of more than $200 million. Considerable efforts to mitigate nutrient, stormwater, and wastewater pollution would need to be made in order to reach these desired scenarios and the value of recreation captures just one part of the possible social values of those mitigation efforts.

## Supplementary Material

Supplement1

## Figures and Tables

**Table 1.** Input data sets and sources

Attribute		Description	Source	References
*Shoreline*				
	Types	Sandy, vegetated, rocky, armored. Exposed/sheltered	NOAA ESI	https://response.restoration.noaa.gov/resources/environmental-sensitivity-index-esi-maps
	Beaches	Beach locations	EPA BEACON	https://www.epa.gov/waterdata/beacon-20-beach-advisory-and-closing-online-notification
*Water*				
	Water quality samples	Chl *a*, Secchi, Enterococcus	National Water Quality Portal (WQP) and Regional Programs	https://www.waterqualitydata.us/, Direct communications
	303(d) Impairments	Impairments to coastal waters from state 303(d) listings	State GIS portals	RI: 2010- https://www.rigis.org/datasets/marine-and-estuarine-waters-ri-integrated-water-quality-monitoring-assessment-2010/explore
				MA: 2014- https://docs.digital.mass.gov/dataset/massgis-data-massdep-2014-integrated-list-waters-305b303d
				NH: 2016- http://nhdes.maps.arcgis.com/apps/webappviewer/index.html?id=aca7a13dced5426aa542c62b1ea10d0c
				CT:2016- https://portal.ct.gov/DEEP/GIS-and-Maps/Data/GIS-DATA#NaturalResourcesManagement
	Remote-sensed clarity	Secchi depth (m)	USGS/NASA Landsat 8 and Google Earth Engine	https://code.earthengine.google.com/a65ff36ae0d22fc4b92c29a7c9178f6f?noload=true
*Land*				
	Impervious surface	Impervious surface as a % of land area	NLCD 2016 Percent Impervious Surface	https://www.mrlc.gov/data/nlcd-2016-percent-developed-imperviousness-conus

**Table 2.** Summary statistics of attributes in the choice set

	Mean	SD	N
Shoreline segment length (m)	289.41	513.67	7232
Beach (yes/no)	0.26	0.44	7243
Sheltered (%)	61	0.46	7232
Sand (%)	25	0.37	7232
Vegetated (%)	48	0.46	7232
Armored (%)	13	0.28	7232
Rocky (%)	13	0.32	7232
Impervious (%)	5.28	5.60	7243
Secchi depth (m)	1.14	0.59	7230
Impaired (yes/no)	0.74	0.44	7243
Enterococcus (CFU or MPN)	58.16	53.84	4812

**Table 3.** Model results

Variable	Description	1	2	3
Travel cost ($)	Round trip cost of travel	−0.051*** (0.001)	−0.051*** (0.001)	−0.050*** (0.002)
Beach	Site includes a beach	1.343*** (0.096)	1.297*** (0.096)	
Size (1000 m)	Straight line length of shoreline	0.595*** (0.033)	0.625*** (0.033)	0.607*** (0.052)
% Sheltered	% of shoreline segment that is sheltered	−0.499*** (0.153)	−0.470*** (0.154)	−0.909*** (0.203)
% Sandy	% of shoreline segment that is sandy	−6.333*** (0.168)	−6.494*** (0.193)	−4.352*** (0.178)
% Rocky	% of shoreline segment that is rocky	−7.946*** (0.323)	−7.967*** (0.338)	−5.497*** (0.426)
% Vegetated	% of shoreline segment that is vegetated	−7.417*** (0.225)	−7.557*** (0.238)	−5.298*** (0.304)
% Armored	% of shoreline segment that is armored	−6.239*** (0.229)	−6.322*** (0.250)	−4.447*** (0.292)
% Impervious	% of nearby area that is impervious cover	−0.098*** (0.012)	−0.091*** (0.012)	−0.090*** (0.017)
Secchi depth (m)	Remote sensing estimate of Secchi depth	0.227*** (0.064)	0.227*** (0.064)	0.231*** (0.076)
Impaired	1/0 existence of 303(d) impairment in adjacent water	0.065 (0.090)	0.117 (0.090)	0.183* (0.104)
CFU or MPN	Bacteria measure by colony-forming units or most probable number		−0.004*** (0.001)	−0.004*** (0.001)
CFU or MPN data available	1/0 to indicate sites with bacteria data		0.287** (0.117)	
WTP for clarity ($/meter)		4.475	4.497 (1.95,6.81)	4.560
WTP for CFU or MPN ($/CFU or MPN)			0.078 (−.10,−.054)	−0.084
N of respondents		706	706	477
AIC		11136.0	11118.6	6657.0
BIC		11279.1	11287.7	6781.7

Note:

* *p* < .1,

** *p* < .05,

*** *p* < .01.

Standard deviation of parameters in parentheses and 95% confidence interval of WTP metrics in brackets. Model 1 excludes bacteria measurements, model 2 includes all water quality variables, and model 3 is fit on a choice set of only trips to beach locations.

**Table 4.** Welfare scenario results

Water quality improvement Cape Cod	Welfare per trip	Welfare per year 80M trips	Present value (r = 3%)
+5% clarity	$0.02 (.007, .026)	$1.3M (.545, 2.06)	$43M (18.2, 68.7)
+ 10% clarity	$0.03 (.014, .052)	$2.6M (1.09, 4.16)	$86M (36.5, 138)
+20% clarity	$0.07 (.027, .106)	$5.2M (2.20, 8.51)	$175M (73.3, 284)
Narragansett Bay			
−5% CFU or MPN	$0.02 (.016, .027)	$1.8M (1.25, 2.20)	$59M (41.7, 73.3)
−10% CFU or MPN	$0.04 (.031, .056)	$3.6M (2.52, 4.45)	$118M (83.9, 148)
−20% CFU or MPN	$0.09 (.064, .114)	$7.2M (5.01, 9.12)	$242M (170, 304)

Note: 95% confidence intervals of welfare metrics in brackets. Results are from simulations of model 2 in Table 3.

## Data Availability

The data and code that support the findings of this study will be openly available in Zenodo at https://doi.org/10.5281/zenodo.6390881.

## Footnotes

1 Following English (2018), we calculated the per mile travel costs as a combination of gas costs, depreciation, and maintenance. Gas and maintenance costs per mile came directly from AAA (2018). We used the difference between the total annual depreciation cost reported for 5,000 miles above and below the baseline of 15,000 mile average annual miles driven in the AAA report. This cost difference is divided by 10,000 miles to create a marginal depreciation expense per mile driven.

2 If the respondent did not answer with their income range, we assigned them the average from our sample – $112,364.

## References

[R1] AAA. 2018. Your Driving Costs. Heathrow, FL: AAA Association Communication.

[R2] Abidoye BO, Herriges JA, and Tobias JL 2012. Controlling for observed and unobserved site characteristics in RUM models of recreation demand. American Journal of Agricultural Economics 94(5): 1070–1093.

[R3] Artell J, Ahtiainen H, and Pouta E 2013. Subjective vs. objective measures in the valuation of water quality. Journal of Environmental Management 130: 288–296. 24095791 10.1016/j.jenvman.2013.09.007

[R4] Bergstrom JC, Dorfman JH, and Loomis JB 2004. Estuary management and recreational fishing benefits. Coastal Management 32(4): 417–432.

[R5] Binkley CS, and Hanemann WM 1978. The Recreation Benefits of Water Quality Improvement: Analysis of Day Trips in Urban Setting. Washington, DC: Environmental Protection Agency, Office of Research and Development, Office of Health and Ecological Effects.

[R6] Bockstael NE, Hanemann WM, and Kling CL 1987a. Estimating the value of water quality improvements in a recreational demand framework. Water Resources Research 23(5): 951–960.

[R7] Bockstael NE, McConnell KE, and Strand IE 1987b. Benefits from Improvements in Chesapeake Bay Water Quality (Volume II of Benefit Analysis using Indirect or Imputed Market Methods). Washington, DC: US Environmental Protection Agency.

[R8] Cape Cod Commission. 2015. 208 Plan: Cape Cod Area Wide Water Quality Management Plan Update. Barnstable, MA: Cape Cod Commission.

[R9] Czajkowski M, Ahtiainen H, Artell J, Budziński W, Hasler B, Hasselström L, Meyerhoff J, Nõmmann T, Semeniene D, Söderqvist T, and Tuhkanen H 2015. Valuing the commons: an international study on the recreational benefits of the Baltic Sea. Journal of Environmental Management 156: 209–217. 25846001 10.1016/j.jenvman.2015.03.038

[R10] Dewitz J 2019. National Land Cover Database (NLCD) 2016 Products (ver. 2.0, July 2020) https://www.mrlc.gov/national-land-cover-database-nlcd-2016. Accessed November 11, 2024.

[R11] Efimova E 2019. A Random Utility Model of Beach Use on the East Coast of the United States: Per-Trip Values and Hypothetical Beach Closures. Newark, DE: University of Delaware Dissertation.

[R12] Egan KJ, Herriges JA, Kling CL, and Downing JA 2009. Valuing water quality as a function of water quality measures. American Journal of Agricultural Economics 91: 106–123.

[R13] English E, von Haefen RH, Herriges J, Leggett C, Lupi F, McConnell K, Welsh M, Domanski A, and Meade N 2018. Estimating the value of lost recreation days from the deepwater horizon oil spill. Journal of Environmental Economics and Management 91: 26–45.

[R14] Environmental Protection Agency. 2021. BEACON 2.0 (Beach Advisory and Closing Online Notification) Available at: https://www.epa.gov/waterdata/beacon-20-beach-advisory-and-closing-online-notification (Accessed October 2021).

[R15] Environmental Protection Agency. 2023a. Indicators: Enterococci. Available at: https://www.epa.gov/national-aquatic-resource-surveys/indicators-enterococci (Accessed August 2023).

[R16] Environmental Protection Agency. 2023b. Recreational Water Quality Criteria and Methods. Available at: https://www.epa.gov/wqc/recreational-water-quality-criteria-and-methods (Accessed August 2023).

[R17] Environmental Systems Research Institute (ESRI). 2020. ArcGIS Release 10.3 Redlands, CA: ESRI.

[R18] Franco SF, and Macdonald JL 2018. Measurement and valuation of urban greenness: remote sensing and hedonic applications to Lisbon, Portugal, Regional Science and Urban Economics 72: 156–180.

[R19] Gómez AG, Ondiviela B, Fernández M, and Juanes JA 2017. Atlas of susceptibility to pollution in marinas. Application to the Spanish coast. Marine Pollution Bulletin 114(1): 239–246. 27641108 10.1016/j.marpolbul.2016.09.009

[R20] Google. 2021. Google Map’s Application Programming Interface (API). Available at: https://cloud.google.com/maps-platform/#products (Accessed October 2021).

[R21] Gorelick N, Hancher M, Dixon M, Ilyushchenko S, Thau D, and Moore R 2017. Google Earth Engine: planetary-scale geospatial analysis for everyone. Remote Sensing of Environment 202: 18–27.

[R22] Greene WH, and Hensher DA 2003. A latent class model for discrete choice analysis: contrasts with mixed logit. Transportation Research Part B: Methodological 37(8): 681–698.

[R23] Hamel K, Lacasse K, and Dalton T 2021. Recreational users’ perceptions of coastal water quality in Rhode Island (USA): Implications for policy development and management. Marine Pollution Bulletin 172: 112810. 34392155 10.1016/j.marpolbul.2021.112810

[R24] Hilger J, and Hanemann M 2006. Heterogeneous Preferences for Water Quality: A Finite Mixture Model of Beach Recreation in Southern California. San Diego, CA: California Sea Grant College Program, Research Completion Reports, University of California.

[R25] Jain M 2020. The benefits and pitfalls of using satellite data for causal inference. Review of Environmental Economics and Policy 14(1): 157–169.

[R26] Jones MP 1996. Indicator and stratification methods for missing explanatory variables in multiple linear regression. Journal of the American Statistical Association 91(433): 222–230.

[R27] Kaoru Y 1995. Measuring marine recreation benefits of water quality improvements by the nested random utility model. Resource and Energy Economics 17(2): 119–136.

[R28] Keiser DA, Kling CL, and Shapiro JS 2019. The low but uncertain measured benefits of US water quality policy. Proceedings of the National Academy of Sciences 116(12): 5262–5269. 10.1073/pnas.1802870115 PMC6431143 30297391

[R29] Kosaka R, and Steinback S 2018. 2012 National Ocean Recreation Expenditure Survey. Silver Spring, Maryland: U.S. Department of Commerce, National Oceanic and Atmospheric Administration, National Marine Fisheries Service.

[R30] Krinsky I, and Robb AL 1986. On approximating the statistical properties of elasticities. The Review of Economics and Statistics 68(4): 715–719.

[R31] Kuwayama Y, Mabee B, and Wulf Tregar S 2017. The consortium for the valuation of applications benefits linked with earth science (VALUABLES). AGU Fall Meeting Abstracts 2017: PA11C–01.

[R32] Lee Z, Shang S, Qi I, Yan J, and Lin G. 2016. A semi-analytical scheme to estimate Secchi disk depth from Landsat 8 measurements. Remote Sensing of Environment 177: 101–106.

[R33] Leeworthy VR 2001. National Survey on Recreation and the Environment (NSRE) Preliminary Estimates from Version 1–6: Coastal Recreation Participation. Silver Spring, MD: US Department of Commerce, National Oceanic and Atmospheric Administration, National Ocean Service, Special Projects Office.

[R34] Leeworthy VR, Bowker JM, and Stone EA 2005. Projected Participation in Marine Recreation: 2005 & 2010. Silver Spring, MD: US Department of Commerce, National Oceanic and Atmospheric Administration.

[R35] Leeworthy VR, and Wiley PC 2001. Current Participation Patterns in Marine Recreation. Silver Spring, MD: National Oceanic and Atmospheric Administration, National Ocean Service.

[R36] Lipton D, and Hicks R 2003. The cost of stress: low dissolved oxygen and economic benefits of recreational striped bass (*Morone saxatilis*) fishing in the Patuxent River. Estuaries 26(2): 310–315.

[R37] Luis KM, Rheuban JE, Kavanaugh MT, Glover DM, Wei J, Lee Z, and Doney SC 2019. Capturing coastal water clarity variability with Landsat 8. Marine Pollution Bulletin 145: 96–104. 31590839 10.1016/j.marpolbul.2019.04.078

[R38] Lupi F and Feather PM, 1998. Using partial site aggregation to reduce bias in random utility travel cost models. Water Resources Research, 34(12), 3595–3603.

[R39] Lupi F, Phaneuf DJ, and von Haefen RH 2020. Best practices for implementing recreation demand models. Review of Environmental Economics and Policy 14(1): 302–323.

[R40] Luxen D, and Vetter C 2011. Real-time routing with OpenStreetMap data. Proceedings of the 19th ACM SIGSPATIAL International Conference on Advances in Geographic Information Systems. New York, NY: Association for Computing Machinery.

[R41] Lyon SF, Merrill NH, Mulvaney KK, and Mazzotta MJ 2018. Valuing coastal beaches and closures using benefit transfer: an application to Barnstable, Massachusetts. Journal of Ocean and Coastal Economics 5(1): 1. 30148207 10.15351/2373-8456.1086 PMC6104649

[R42] Mandelbrot BB 1982. The Fractal Geometry of Nature. New York: WH Freeman.

[R43] Mazzotta MJ, Merrill NH, and Mulvaney KK 2022. Coastal recreation in southern New England: Results from a regional survey. Journal of Ocean and Coastal Economics. 9(1): 1. 36275927 10.15351/2373-8456.1152 PMC9580342

[R44] Merrill N, Sawyer J, Erban L, Mulvaney K, Mazzotta M, and Keith D 2021. Water Quality Data Synthesis of New England Coastal Waters for Social Science Endpoints. 12th National Water Quality Monitoring Conference. April 2021. https://www.nalms.org/2021nmc/.

[R45] Merrill NH, Atkinson SF, Mulvaney KK, Mazzotta MJ, and Bousquin J 2020. Using data derived from cellular phone locations to estimate visitation to natural areas: an application to water recreation in New England, USA. PloS One 15(4): e0231863. 32352978 10.1371/journal.pone.0231863 PMC7192446

[R46] Merrill NH, Piscopo AN, Balogh S, Furey RP, and Mulvaney KK 2021. When, where, and how to intervene? Tradeoffs between time and costs in coastal nutrient management. Journal of the American Water Resources Association 57(2): 328–343. 35153467 10.1111/1752-1688.12897 PMC8827406

[R47] Mulvaney KK, Atkinson SF, Merrill NH, Twichell JH, and Mazzotta MJ 2020. Quantifying recreational use of an estuary: a case study of Three Bays, Cape Cod, USA. Estuaries and Coasts 43(1): 7–22. 32280317 10.1007/s12237-019-00645-8 PMC7147807

[R48] Murdock J 2006. Handling unobserved site characteristics in random utility models of recreation demand. Journal of Environmental Economics and Management 51(1): 1–25.

[R49] National Oceanic and Atmospheric Administration. 2021a. Types of Recreational Fishing Surveys. Available at https://www.fisheries.noaa.gov/recreational-fishing-data/types-recreational-fishing-surveys (Accessed October 2021).

[R50] National Oceanic and Atmospheric Administration. 2021b. Environmental Sensitivity Index (ESI) Maps and Data. Available at https://response.restoration.noaa.gov/resources/environmental-sensitivity-index-esi-maps (Accessed October 2021).

[R51] National Oceanic and Atmospheric Administration. 2021c. What percentage of the American population lives near the coast? Available at https://oceanservice.noaa.gov/facts/population.html (Accessed October 2021).

[R52] NBEP. Narragansett Bay Estuary Program. 2017. The state of Narragansett Bay and its watershed—technical report. Available at http://nbep.org/01/wp-content/uploads/2017/09/State-of-Narragansett-Bay-and-Its-Watershed.pdf (Accessed October 2021).

[R53] Oh C-O, Dixon AW, Mjelde JW, and Draper J 2008. Valuing visitors’ economic benefits of public beach access points. Ocean & Coastal Management 51(12): 847–853.

[R54] Opaluch JJ, Grigalunas T, Diamantides J, Mazzotta M, and Johnston R 1999. Recreational and Resource Economic Values for the Peconic Estuary System. Jackson, MS: Economic Analysis Inc.

[R55] Parsons GR, and Needelman MS 1992. Site aggregation in a random utility model of recreation. Land Economics 68(4): 418–433.

[R56] Parsons GR 2003. The travel cost model. In A Primer on Nonmarket Valuation. Dordrecht: Springer.

[R57] Parsons GR 2017. Travel cost models. In A Primer on Nonmarket Valuation. Dordrecht: Springer.

[R58] Parsons GR, Kang AK, Leggett CG, and Boyle KJ 2009. Valuing beach closures on the Padre Island National Seashore. Marine Resource Economics 24: 213–235.

[R59] Perry ES, Smith SN, and Mulvaney KK 2020. Designing solutions for clean water on Cape Cod: engaging communities to improve decision making. Ocean & Coastal Management 183: 104998. 10.1016/j.ocecoaman.2019.104998 PMC8193831 34121820

[R60] Phaneuf DJ 2002. A random utility model for total maximum daily loads: estimating the benefits of watershed-based ambient water quality improvements. Water Resources Research 38(11): 36–31.

[R61] Phaneuf DJ, and Smith VK 2005. Recreation demand models. Handbook of Environmental Economics 2: 671–761.

[R62] QGIS.org. 2021. QGIS Geographic Information System. QGIS Association. Available at http://www.qgis.org (Accessed October 2021).

[R63] R Core Team. 2013. R: A Language and Environment for Statistical Computing. Vienna, Austria: R Core Team.

[R64] Read EK, Carr L, De Cicco L, Dugan HA, Hanson PC, Hart JA, Kreft J, Read JS, and Winslow LA 2017. Water quality data for national-scale aquatic research: the water quality portal. Water Resources Research 53(2): 1735–1745.

[R65] Sarrias M 2016. Discrete choice models with random parameters in R: the Rchoice package. Journal of Statistical Software 74(1): 1–31.

[R66] Sprague LA, Oelsner GP, and Argue DM 2017. Challenges with secondary use of multi-source water-quality data in the United States. Water Research 110: 252–261. 28027524 10.1016/j.watres.2016.12.024

[R67] Stedman RC, Connelly NA, Heberlein TA, Decker DJ, and Allred SB 2019. The end of the (research) world as we know it? Understanding and coping with declining response rates to mail surveys. Society & Natural Resources 32(10): 1139–1154.

[R68] Strand IE, and McConnell KE 1985. Measuring economic benefits from Chesapeake Bay water quality improvements. Estuaries 8(2): 51.

[R69] Train KE 1998. Recreation demand models with taste differences over people. Land Economics 74(2): 230–239.

[R70] Twichell J, Mulvaney K, Merrill NH, and Bousquin J 2022. Geographies of dirty water: landscape-scale inequities in coastal access in Rhode Island. Frontiers in Marine Science 8: 760684. 10.3389/fmars.2021.760684 PMC8903087 35273967

[R71] U.S. Census Bureau. 2021. 2014–2019 American Community Survey 5-year Public Use Microdata Samples. Available at https://data.census.gov/cedsci/ (Accessed October 2021).

[R72] von Haefen RH, and Domanski A 2018. Estimation and welfare analysis from mixed logit models with large choice sets. Journal of Environmental Economics and Management 90: 101–118.

[R73] Wolf D, and Kemp T 2021. Convergent validity of satellite and Secchi disk measures of water clarity in hedonic models. Land Economics 97(1): 39–58.

[R74] Zhang J, Phaneuf DJ, and Schaeffer BA 2022. Property values and cyanobacterial algal blooms: evidence from satellite monitoring of Inland Lakes. Ecological Economics 199: 107481.

