# Supplementary material for: The value of water quality for coastal recreation in New England, USA
Source: Agric Resour Econ Rev. Author manuscript; Available in PMC 2025 Dec 1. (PMC12180910; doi:10.1017/age.2024.12)
Supplement: Supplement1 [file NIHMS2040284-supplement-Supplement1.pdf]

Supplementary Materials for

The Value of Water Quality for Coastal Recreation in New England, USA

Shoreline Segments

NOAA's Environmental Sensitivity Index (ESI) data included up to three shoreline types for each segment, representing landward to seaward types. Their simplified "general_symbol" types selected the most environmentally sensitive of these. We created a crosswalk to develop general categories to indicate the type most relevant to recreational choices. For each shoreline segment, we recoded the ESI "general shoreline types" to more general "New England Water Quality (NEWQ)" categories of armored, rocky and steep, beach, or vegetated. We then merged the segments based on these simplified NEWQ categories, combining like-type adjacent shorelines, resulting in 17,665 shoreline segments from Connecticut to Maine (see TABLE S1 for ESI shoreline type crosswalk). We conducted processing using ArcGIS ArcPy scripting functionality (ESRI 2020).

We created an exposed/sheltered attribute for the NEWQ segments ("NEWQ shoreline segments"; Figure 1) based on the presence of "exposed" or "sheltered" in the ESI "shoretypes" descriptions for the three landward to seaward shoreline types. NOAA's type 1 and 2 shoretypes were classified as exposed; shoretypes 8, 9, and 10 were classified as sheltered.

TABLE S1: ESI to NEWQ Shoreline Type Crosswalk

Generalized ESI Type	NEWQ shoreline type*
1: Armored	1
1: Armored/2: Rocky and Steep Shorelines (Bedrock/Sand/Clay)	1
1: Armored/2: Rocky and Steep Shorelines (Bedrock/Sand/Clay)/3: Beaches (Sand/Gravel)	3
1: Armored/2: Rocky and Steep Shorelines (Bedrock/Sand/Clay)/4: Flats (Mud/Sand)	1
1: Armored/3: Beaches (Sand/Gravel)	3
1: Armored/3: Beaches (Sand/Gravel)/2: Rocky and Steep Shorelines (Bedrock/Sand/Clay)	3
1: Armored/3: Beaches (Sand/Gravel)/4: Flats (Mud/Sand)	3
1: Armored/4: Flats (Mud/Sand)	1
1: Armored/5: Vegetated (Grass/Marsh/Mangroves/Scrub-Shrub)	1
1: Armored/5: Vegetated (Grass/Marsh/Mangroves/Scrub-Shrub)/3: Beaches (Sand/Gravel)	1
1: Armored/5: Vegetated (Grass/Marsh/Mangroves/Scrub-Shrub)/4: Flats (Mud/Sand)	1
2: Rocky and Steep Shorelines (Bedrock/Sand/Clay)	2
2: Rocky and Steep Shorelines (Bedrock/Sand/Clay)/3: Beaches (Sand/Gravel)	3
2: Rocky and Steep Shorelines (Bedrock/Sand/Clay)/3: Beaches (Sand/Gravel)/4: Flats (Mud/Sand)	3
2: Rocky and Steep Shorelines (Bedrock/Sand/Clay)/4: Flats (Mud/Sand)	2
2: Rocky and Steep Shorelines (Bedrock/Sand/Clay)/5: Vegetated (Grass/Marsh/Mangroves/Scrub-Shrub)	2
3: Beaches (Sand/Gravel)	3
3: Beaches (Sand/Gravel)/1: Armored	3
3: Beaches (Sand/Gravel)/1: Armored/4: Flats (Mud/Sand)	3
3: Beaches (Sand/Gravel)/2: Rocky and Steep Shorelines (Bedrock/Sand/Clay)	3
3: Beaches (Sand/Gravel)/2: Rocky and Steep Shorelines (Bedrock/Sand/Clay)/4: Flats (Mud/Sand)	3

3: Beaches (Sand/Gravel)/4: Flats (Mud/Sand)	3
3: Beaches (Sand/Gravel)/5: Vegetated (Grass/Marsh/Mangroves/Scrub-Shrub)	3
3: Beaches (Sand/Gravel)/5: Vegetated (Grass/Marsh/Mangroves/Scrub-Shrub)/4: Flats (Mud/Sand)	3
5: Vegetated (Grass/Marsh/Mangroves/Scrub-Shrub)	5
5: Vegetated (Grass/Marsh/Mangroves/Scrub-Shrub)/1: Armored	5
5: Vegetated (Grass/Marsh/Mangroves/Scrub-Shrub)/1: Armored/3: Beaches (Sand/Gravel)	5
5: Vegetated (Grass/Marsh/Mangroves/Scrub-Shrub)/1: Armored/4: Flats (Mud/Sand)	5
5: Vegetated (Grass/Marsh/Mangroves/Scrub-Shrub)/2: Rocky and Steep Shorelines (Bedrock/Sand/Clay)	5
5: Vegetated (Grass/Marsh/Mangroves/Scrub-Shrub)/2: Rocky and Steep Shorelines (Bedrock/Sand/Clay)/3: Beaches (Sand/Gravel)	5
5: Vegetated (Grass/Marsh/Mangroves/Scrub-Shrub)/2: Rocky and Steep Shorelines (Bedrock/Sand/Clay)/4: Flats (Mud/Sand)	5
5: Vegetated (Grass/Marsh/Mangroves/Scrub-Shrub)/3: Beaches (Sand/Gravel)	3
5: Vegetated (Grass/Marsh/Mangroves/Scrub-Shrub)/3: Beaches (Sand/Gravel)/2: Rocky and Steep Shorelines (Bedrock/Sand/Clay)	5
5: Vegetated (Grass/Marsh/Mangroves/Scrub-Shrub)/3: Beaches (Sand/Gravel)/4: Flats (Mud/Sand)	5
5: Vegetated (Grass/Marsh/Mangroves/Scrub-Shrub)/4: Flats (Mud/Sand)	5

*NEWQ Types: 1 = armored; 2=rocky/steep; 3=beach; 4=flats; 5=vegetated

EPA’s BEACON system defines beach location in their Reach Address Database (RAD). We first combined the beach lines (RAD lines) where they overlapped, then joined them spatially to the NEWQ shorelines. Lastly, we dissolved the shoreline segments where there were beaches lines occurring across multiple NEWQ shorelines, to maintain beaches as single units, with the other shoreline attributes summarized by percent of the segment with each shoreline type.

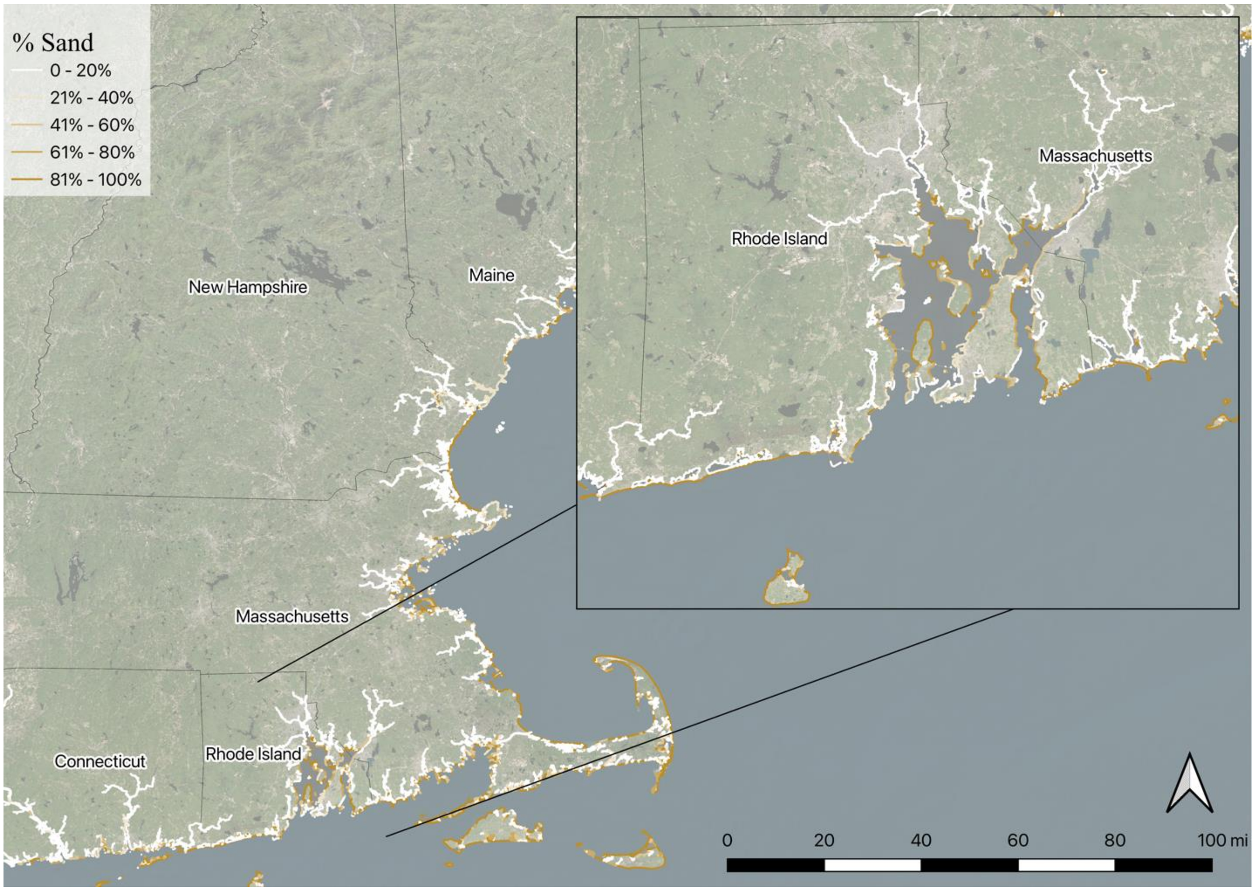

Figure S2: NEWQ Sandy Shorelines

Travel time and distance

Table S2 shows the one-way distance traveled in miles and time traveled in minutes for day trips. The table shows both the self-reported distance and time and the calculated distance and time from the respondent's home census block to the chosen coastal recreation location. Respondents' unweighted mean reported distance and time are slightly less than the calculated mean distance and time but are close.

TABLE S2: Reported and Measured One-Way Distance and Time Traveled for Day Trips

One-Way Distance and Time Traveled	Mean	Linearized Std. Error	95% Confidence Interval	
Reported distance (mi)	30.2	1.53	27.18	33.18
Measured distance (mi)	36.8	1.49	33.88	39.73
Reported time (min)	56.4	3.18	50.11	62.61
Measured time (min)	59.0	3.33	52.48	65.57

TABLE S3: Estimated Days Per Year for Survey Sample Area

Season	Mean Days*	Number of Participants**	Estimated Total Days
Spring (March, April, May)	9.0	3,401,893	30,449,820

Summer (June, July, August)	18.8	4,252,072	80,066,906
Fall (September, October, November)	10.2	3,537,166	35,946,516
Winter (December, January, February)	3.3	2,981,586	9,822,978
12-month total	36.8	4,629,453	170,498,066

*Days were truncated to remove values >95th percentile; seasonal responses include zeros; for total year estimates, zeros were entered as missing values.
**Participants include people aged 18 and older

Economic Methods

Analysis of Revealed Preference Data

We analyzed the revealed preference survey data using a random utility model (RUM). Bockstael et al. (1987) wrote the seminal work applying the RUM to recreation demand in the context of recreation and water quality. The RUM approach has been applied extensively within revealed preference research and allows for well-defined welfare measures (e.g., willingness to pay) to be derived from observed recreation choice behavior using the travel cost method (Phaneuf and Smith 2005; Parsons 2003).

Following Phaneuf and Smith (2005) and Parsons (2003 and 2017) closely, the RUM approach assumes that individuals choose where to recreate by choosing the choice alternative that gives the highest utility. The individual is assumed to face a set of I possible sites for a trip. The sites might be beaches, parks, boat ramps, or other coastal public access points (Parsons 2003). Each site i (i=1, 2…I) is assumed to give the individual, n, some utility U_{in} on a given choice occasion.

Following standard random utility theory, utility is assumed known to the respondent, but stochastic from the perspective of the researcher, such that:

$$(S1) \quad U_{in}(\cdot) = U(\boldsymbol{X_i}, \boldsymbol{D_n}, T_{in}) = v(\boldsymbol{X_i}, \boldsymbol{D_n}, T_{in}) + \varepsilon_{in}$$

where:

$\boldsymbol{X_i}$	=	a vector of variables describing attributes of recreation site *i*
$\boldsymbol{D_n}$	=	a vector of demographic and other attributes of the respondent *n*
T_{in}	=	the cost of choosing site *i* for respondent *n* (the travel cost)
$v(\cdot)$	=	a function representing the empirically estimable component of utility
ε_{in}	=	the stochastic or unobservable component of utility

Standard RUMs are based on the probability that a respondent's utility from site i, $U_{in}(\cdot)$, exceeds the utility from alternative site j, $U_{jn}(\cdot)$, for all potential sites j≠i considered by the respondent. The RUM presumes that the respondent considers the utility that would result from each recreational site choice i, and chooses the site that provides the highest utility, or:

$$(S2) \quad V_n = max(U_{1n}, U_{2n}, \ldots., U_{In})$$

Changes in this per-trip utility, V_n, can be used to value a loss or gain from site access (simulated as a removal or addition of a site to the choice set) as well as for changes in site quality, such as water quality (Parsons 2017). Suppose water quality at sites 2 and 3 is improved through a water quality improving project. If so, trip utility for person n becomes:

$$(S3) \quad V_n^{clean} = max(U_{1n}, U_{2n}^{*}, U_{3n}^{*}, \ldots, U_{In})$$

Where U_{2n}^* and U_{3n}^* are the higher utility due to the improved water quality. Trip utility increases from V_n to V_n^{clean}. Change in utility is monetized by dividing the change by the negative of the coefficient on trip cost (α), which is a measure of the marginal utility of income. This creates the welfare effects (w_n^{clean}) in monetary terms for changes in trip utility. These are estimated changes in welfare represented on a per-trip, per-person basis (Parsons 2017).

$$(S4) \quad w_n^{clean} = \frac{(V_n^{clean} - V_n)}{-\alpha}$$

Econometric specification

Consider $v(\cdot)$, the estimable component of utility in (S1) in a simple linear form most common in the literature (Phaneuf 2005),

$$\text{(S5)} \qquad v_{in} = \alpha T_{in} + \boldsymbol{\zeta X_i}$$

where:

α	=	the travel cost coefficient, or marginal utility of the cost of the trip
T_{in}	=	the cost of choosing site *i* for respondent *n* (the travel cost)
$\boldsymbol{\zeta}$	=	a vector of coefficients for site attributes, $\mathbf{X}_i$

The response variable collected in the survey is binary (1 if the respondent went to site *i*, 0 otherwise). We used a logistic (logit) regression to model this process. This assumes the log of the odds ratio of visiting a site is a linear function of covariates and is solved using maximum likelihood, fitting parameters that make the outcomes observed most likely. The likelihood function is derived from assuming the errors in equation (S5) are from an i.i.d extreme value distribution (see Parsons 2017).

Below, we replace v_{in} with the more specific logit function to make clear the connection between utility, the computational methods and the choice probabilities. We estimated a conditional logit model specification, as follows:

$$(S6) \quad logit(p_{in}) = \alpha T_{in} + \boldsymbol{\zeta X_i}$$

where:

p_{in}	=	the probability that respondent *n* chooses site *i*
$logit(p_{in})$	=	the log of the odds ratio of respondent *n* visiting site *i*
α	=	the travel cost coefficient, or marginal utility of the cost of the trip
T_{in}	=	cost of choosing site *i* for respondent *n*, the travel cost;
$\boldsymbol{\zeta}$	=	a vector of coefficients for site attributes, $\mathbf{X}_i$;

The logit function:

$$(S7) \quad logit(p_{in}) = \ln\left(\frac{p_{in}}{1 - p_{in}}\right)$$

As shown in (Train 2009) for the logit model, the probability that respondent *n* chooses site *i,* where *I* is all alternatives in the choice set, is:

$$(S8) \quad p_{in} = \frac{e^{\alpha T_{in} + \zeta X_i}}{\sum_{i=1}^{I} e^{\alpha T_{in} + \zeta X_i}}$$

where:

I	=	all site alternatives in the choice set

Mixed (Random Parameter) Logit Model

The mixed (random parameter) logit model is a variation of the more commonly used logit presented in the manuscript. The mixed logit model assumes each respondent's parameters may come from a distribution instead of a single value, allowing it to capture more flexible choice patterns than the standard logit model. However, mixed logit requires assuming distributions and fitting additional parameters (distributional parameters of each covariate's coefficient) and making choices about correlations between the coefficients (von Haefen & Domanski 2010).

We fit a mixed logit model for the whole choice set. Using a mixed logit adds significant computational burden to solve even on small choice sets. The full model run on the whole choice set took 50+ hours to converge on a desktop computer with 192GB of RAM and 2x 2.6 GHz processors. We assumed normally distributed coefficients in a mixed logit model using 20 Halton draws. We used R version 4.1.1 and the Rchoice package to fit the model. We created the welfare metrics and scenario results below.

TABLE S4: Mixed logit models

Variable	Description	Coefficients	Standard Deviation of Coefficients
Travel Cost ($)	Round trip cost of travel	-0.077.*** (0.003)	0.024.*** (0.002)
Beach	Site includes a beach	1.313.*** (0.098)	0.077 (0.169)
Size (1000m)	Straight line length of shoreline	0.628.*** (0.037)	0.013 (0.098)
% Sheltered	% of shoreline segment that is sheltered	-0.482.*** (0.155)	0.027 (0.223)
% Sandy	% of shoreline segment that is sandy	-6.105.*** (0.207)	0.179 (0.200)
% Rocky	% of shoreline segment that is rocky	-7.528.*** (0.354)	0.061 (0.890)
% Vegetated	% of shoreline segment that is vegetated	-7.149.*** (0.249)	0.167 (0.342)
% Armored	% of shoreline segment that is armored	-5.913.*** (0.264)	0.141 (0.422)
% Impervious	% of nearby area that is impervious cover	-0.101.*** (0.017)	0.035.** (0.014)
Secchi depth (m)	Remote sensing estimate of Secchi depth	0.210.*** (0.071)	0.132.* (0.069)
Impaired	1/0 existence of 303(d) impairment in adjacent water	0.116 (0.092)	0.027 (0.105)

Variable	Description	Coefficients	Standard Deviation of Coefficients
CFU or MPN	Bacteria measure by colony forming units or most probable number	-0.005.*** (0.001)	0.003.*** (0.001)
CFU or MPN data available	1/0 to indicate sites with bacteria data	0.309.*** (0.119)	0.075 (0.082)
WTP for clarity ($/meter)		$2.719	
WTP for CFU or MPN ($/CFU or MPN)		$-0.061	
N of respondents		706	

Note: * p < .1, ** p < .05, *** p < .01

TABLE S5: Welfare Scenario Results for Full Mixed Logit

Water Quality Improvement Cape Cod	Welfare per Trip	Welfare per Year 80M Trips	Present Value (r=3%)
+5% clarity	$0.01	$.61	$20M
+10% clarity	$0.02	$1.2M	$41M
+20% clarity	$0.03	$2.5M	$82M
Narragansett Bay			
-5% CFU or MPN	$0.02	$1.6M	$53M
-10% CFU or MPN	$0.04	$3.2M	$108M
-20% CFU or MPN	$0.08	$6.6M	$220M

The mixed logit model produced lower welfare values than the standard logit model. Both clarity and bacteria conditions had a smaller impact using mixed logit which played through to the scenario results. The significant standard deviation of the individual-level coefficients for clarity and bacteria (the mixed part of the mixed logit) implies there may be variability in how respondents value water quality in these dimensions.

Alternative Specific Constants Models

RUM models for recreation demand may suffer from unobserved variable bias and heterogeneity among respondents, which can affect the travel cost coefficient and coefficients of site attributes. To address

this, two-stage models using alternative specific constants (ASC) in the first stage along with the travel cost have been proposed. In the second stage, these ASCs are regressed against the site attributes (Murdock 2006).

With our large choice set (7k+ segments), ASC models required aggregation or simplification of the choice set to be computationally feasible. Additionally, ASC methods require that alternatives are chosen at least once, preferably more, to numerically fit the constants, and we had many sites that were not chosen and many that were chosen by only one person. A few alternative methods have been proposed when choice sets are sparsely chosen (Melstrom and Jaysekera 2017). To enable estimation of an ASC model, we created an aggregated choice set based on the zip codes of the shoreline segments (where each zip code became a single site), a second choice set including only those shoreline segments chosen once or more, and a third choice set of only the chosen (at least once) shoreline segments.

For the zipcode model, we dropped zip codes with no visits, leaving a choice set of 138 chosen zip codes, which allowed for a feasible application of ASC models. To estimate this aggregated choice set model, we aggregated water quality and site attributes using averages across segments within a zip code and sums for number of beaches and length of shoreline. For the chosen segment only choice set, we left the attributes as they were in the full model.

The first stage of the ASC model is a logit model:

$$(S9) \quad v_{in} = asc_i + \alpha \, T_{in}$$

where:

asc_i	=	the alternative specific constant for site *i*
α	=	the travel cost coefficient, or marginal utility of the cost of the trip
T_{in}	=	cost of choosing site *i* for respondent *n*, the travel cost

The second stage of the ASC model is a linear regression:

$$(S10) \quad asc_i = \boldsymbol{\zeta X_i} + \varepsilon_i$$

where:

$\boldsymbol{\zeta}$	=	a vector of coefficients for site attributes, $\mathbf{X}_i$
ε_i	=	error term

We used R version 4.1.1 and the mlogit package to fit the first stage and the lm function for the second stage linear regression (R Core Team 2013, Croissant 2012). We simulated the welfare scenarios in R.

TABLE S6: . Stage 1 of ASC Models

Variable	Description	zipcodes	chosen
Travel Cost ($)	Round trip cost of travel	-0.061*** (0.002)	-.091***(0.003)
N of respondents		731	729

Note: * p < .1, ** p < .05, *** p < .01

TABLE S7: Stage 2 of ASC Models

Variable	Description	zipcodes	chosen
# of Beaches	Number of beaches	0.015** (0.009)	
Beach	Site includes a beach		0.127 (0.152)
Size (1000m)	Straight line length of shoreline	0.041.*** (0.010)	0.149.** (0.066)
% Sheltered	% of shoreline segment that is sheltered	-0.719 (0.665)	-0.065 (0.232)
% Sandy	% of shoreline segment that is sandy	0.498 (0.823)	0.350 (0.306)
% Rocky	% of shoreline segment that is rocky	0.413 (1.023)	-0.652 (0.502)
% Armored	% of shoreline segment that is armored	-1.097 (0.722)	-0.424 (0.311)
% Impervious	% of nearby area that is impervious cover	-0.072 (0.047)	0.002 (0.018)
Secchi depth (m)	Remote sensing estimate of Secchi depth	-0.167 (0.361)	0.065 (0.113)

Variable	Description	zipcodes	chosen
Impaired	1/0 existence of 303(d) impairment in adjacent water	-0.382 (0.353)	-0.445.*** (0.151)
CFU or MPN	Bacteria measure by colony forming units or most probable number	-0.006.*** (0.002)	-0.006.*** (0.001)
CFU or MPN data available	1/0 to indicate sites with bacteria data		0.078 (0.183)
N		138	394
R^2		0.54	0.19

Note: * p < .1, ** p < .05, *** p < .01

To create a welfare scenario from the ASC models, we used the zipcode version. We used the same coastline segment scenario attribute adjustments as in the non-ASC model and aggregated the change to the zip-code level in the same way we created the zip-code level attributes for the baseline model. First, these attribute changes are simulated through the second-stage ASC regression (equation S10), predicting new ASCs. These constants are then simulated through the first stage (equation S9) to create new respondent and alternative choice utilities. We calculated the welfare changes from there in the same manner as before using the logsum approach and equation 3. We only created welfare scenarios for the bacteria changes because clarity was insignificant in the ASC model.

TABLE S8: . Welfare from ASC zipcode model

Water Quality Improvement	Welfare Per Trip	Welfare per Year<br>80M Trips	Present Value (r=3%)
Narragansett Bay			
-5% CFU or MPN	$0.03	$2.5M	$82M

Water Quality Improvement	Welfare Per Trip	Welfare per Year<br>80M Trips	Present Value (r=3%)
Narragansett Bay			
-10% CFU or MPN	$0.06	$5.0M	$166M
-20% CFU or MPN	$0.13	$10.2M	$339M

Water clarity was insignificant in both of the ASC models. Bacteria conditions had a larger influence in the ASC model, resulting in larger welfare scenario results for Narragansett Bay. The formulation of the ASC model required significant aggregation of our choice set as compared to our preferred standard conditional logit with the fully disaggregated choice set, taking advantage of water quality variation at the shoreline segment level. In order to estimate the ASC model, we had to make simplifying steps that abstract away from the choice set design and water quality variation we sought to study.

Since we had 7k+ sites in the original model, we grouped them by zip code, which is roughly grouping to towns in New England, creating averages of the segments for each zip code. In some cases, this resulted in combining and averaging conditions of estuary and open ocean waters. We also had to drop any unchosen zip codes, since ASC models need sites to be chosen, at least a few times, to computationally fit the constants; alternatively, one could estimate a more complicated hurdle type model that allows for zero trip options. Our aggregation resulted in 138 ASCs (or 394 in the chosen site only model), which is in line with the maximum ASCs found in the literature. However, neither of these options is a preferred approach to creating choice sets, with bias issues created by aggregation and by elimination of choices and arbitrary groupings. We did not prefer dropping relevant alternatives in the chosen site only model for the same reason, as this is not considered good choice set design practice (Parsons 2000; Lupi et al 2020). It is unclear if the proposed benefits of the ASC models outweigh the downsides it imposed for this survey collection and attribute dataset.

REFERENCES

Bockstael, N.E., Hanemann, W.M. and Kling, C.L., 1987. Estimating the value of water quality improvements in a recreational demand framework. Water Resources Research 23(5): 951-960.

Croissant, Y., 2012. Estimation of multinomial logit models in R: The mlogit Packages. R package version 0.2-2. URL: http://cran. r-project.org/web/packages/mlogit/vignettes/mlogit.pdf.

Lupi, F., Phaneuf, D.J., and and von Haefen, R.H., 2020. Best practices for implementing recreation demand models. Review of Environmental Economics and Policy 14(1): 302-323.

McConnell, K. E. and Tseng, W. C., 1999. Some preliminary evidence on sampling of alternatives with the random parameters logit. Marine Resource Economics 14(4): 317-332.

Melstrom, R.T. and Jayasekera, D.H., 2017. Two-stage estimation to control for unobservables in a recreation demand model with unvisited sites. Land Economics 93(2): 328-341.

Murdock, J., 2006. Handling unobserved site characteristics in random utility models of recreation demand. Journal of Environmental Economics and Management, 51(1): 1-25.

Nerella, S., & Bhat, C. R. 2004. Numerical analysis of effect of sampling of alternatives in discrete choice models. Transportation Research Record 1894(1): 11-19.

Parsons, G.R., 2003. The travel cost model. In A primer on nonmarket valuation (pp. 269-329). Springer, Dordrecht.

Parsons, G.R., Plantinga, A.J. and Boyle, K.J. 2000. Narrow choice sets in a random utility model of recreation demand. Land Economics 76(1): 86-99

Parsons, G.R., 2017. Travel cost models. A primer on nonmarket valuation, pp.187-233.

Phaneuf, D.J. and Smith, V.K., 2005. Recreation demand models. Handbook of Environmental Economics, 2: 671-761.

R Core Team, 2013. R: A language and environment for statistical computing.

Train, K. E. 2009. *Discrete Choice Methods with Simulation*. Cambridge University Press.

von Haefen, R. H. & Domanski, A. 2018. Estimation and welfare analysis from mixed logit models with large choice sets. Journal of Environmental Economics and Management 90(C): 101-118.
